# Creating a specialist protein resource network: a meeting report for the protein bioinformatics and community resources retreat

**DOI:** 10.1093/database/bav063

**Published:** 2015-07-11

**Authors:** Patricia C. Babbitt, Pantelis G. Bagos, Amos Bairoch, Alex Bateman, Arnaud Chatonnet, Mark Jinan Chen, David J. Craik, Robert D. Finn, David Gloriam, Daniel H. Haft, Bernard Henrissat, Gemma L. Holliday, Vignir Isberg, Quentin Kaas, David Landsman, Nicolas Lenfant, Gerard Manning, Nozomi Nagano, Narayanaswamy Srinivasan, Claire O’Donovan, Kim D. Pruitt, Ramanathan Sowdhamini, Neil D. Rawlings, Milton H. Saier, Joanna L. Sharman, Michael Spedding, Konstantinos D. Tsirigos, Ake Vastermark, Gerrit Vriend

**Affiliations:** ^1^Department of Bioengineering and Therapeutic Sciences and California Institute for Quantitative Biosciences, University of California San Francisco, 1700 4th Street, San Francisco, CA 94158, USA,; ^2^Department of Computer Science and Biomedical Informatics, University of Thessaly, Papasiopoulou 2-4, Lamia, 35100, Greece,; ^3^SIB—Swiss Institute of Bioinformatics, CMU, 1 rue Michel Servet, 1211 Geneva 4, Switzerland,; ^4^European Molecular Biology Laboratory, European Bioinformatics Institute (EMBL-EBI), Wellcome Trust Genome Campus, Hinxton, Cambridge CB10 1SD, UK,; ^5^INRA, UMR866 Dynamique Musculaire et Métabolisme, F-34000 Montpellier, France,; ^6^Razavi Newman Center for Bioinformatics, Salk Institute, 10010 North Torrey Pines Rd., La Jolla, CA 92037, USA,; ^7^Bioinformatics & Computational Biology, Genentech, 1 DNA Way, South San Francisco, CA 94080, USA,; ^8^Queensland Bioscience Precinct, 306 Carmody Rd, Building 80, The University of Queensland, Australia,; ^9^Department of Drug Design and Pharmacology, University of Copenhagen, Jagtvej 162, 2100 København Ø, Denmark,; ^10^National Center for Biotechnology Information, National Library of Medicine, National Institutes of Health, Building 38 A, 8600 Rockville Pike, Bethesda, MD 20894, USA,; ^11^Architecture et Fonction des Macromolécules Biologiques, CNRS, Aix-Marseille Université, 13288 Marseille, France,; ^12^Department of Biological Sciences, King Abdulaziz University, Jeddah, Saudi Arabia,; ^13^CMBI, Raboudumc, Geert Grootplein Zuid 26-28, 6525 GA Nijmegen, The Netherlands,; ^14^Biotechnology Research Institute for Drug Discovery, National Institute of Advanced Industrial Science and Technology (AIST), 2-4-7 Aomi, Koto-ku, Tokyo 135-0064, Japan,; ^15^Molecular Biophysics Unit, Indian Institute of Science, Bangalore 560 012, India,; ^16^National Centre for Biological Sciences, TIFR, GKVK Campus, Bangalore 560 065, India,; ^17^Department of Molecular Biology, University of California at San Diego, La Jolla, CA 92093-0116, USA,; ^18^Centre for Integrative Physiology, University of Edinburgh, Hugh Robson Building, George Square, Edinburgh EH8 9XD, UK,; ^19^Spedding Research Solutions, 6 Rue Ampere, 78110 Le Vesinet, France and; ^20^Department of Biochemistry and Biophysics, Science for Life Laboratory, Swedish E-Science Research Center, Stockholm University, Box 1031, 17121 Solna, Sweden

## Abstract

During 11–12 August 2014, a Protein Bioinformatics and Community Resources Retreat was held at the Wellcome Trust Genome Campus in Hinxton, UK. This meeting brought together the principal investigators of several specialized protein resources (such as CAZy, TCDB and MEROPS) as well as those from protein databases from the large Bioinformatics centres (including UniProt and RefSeq). The retreat was divided into five sessions: (1) key challenges, (2) the databases represented, (3) best practices for maintenance and curation, (4) information flow to and from large data centers and (5) communication and funding. An important outcome of this meeting was the creation of a Specialist Protein Resource Network that we believe will improve coordination of the activities of its member resources. We invite further protein database resources to join the network and continue the dialogue.

## Introduction

### Motivation for the meeting

Many databases exist that provide information to the scientific community to enable the understanding of particular classes of proteins. For example, the CAZy database (1) provides detailed information about carbohydrate enzymes, and the TCDB database provides the classification and descriptions of transporter proteins (2). These databases are usually run by a world-leading expert, and most of these databases have a main focus on curating fundamental molecular data about proteins, often linking sequence, structural and functional features relevant to a broad range of fields including molecular biology, biomedicine and biotechnology. Because each resource has developed to serve a particular community of researchers, a variety of tools, techniques and philosophies have evolved to best serve their communities. Often the groups involved in running these resources are well engaged with their community of biologists but are not well connected to those running similar databases for different communities. As new data become available, and these data are integrated for greater impact and predictive power, we saw value in bringing these diverse community resources together to explore how interactions with each other could improve all and contribute more effectively to serving our users.

### Participation

**Figure 1.**
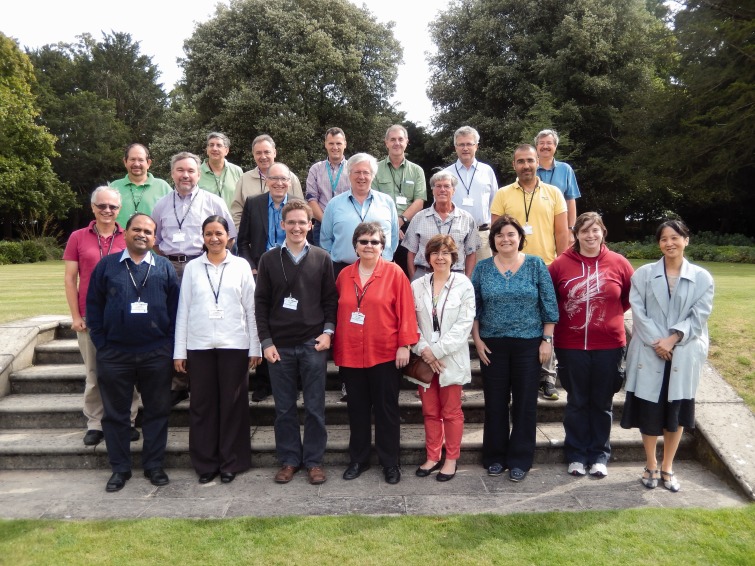
Group photo of the participants at the Protein Bioinformatics and Community Resources Retreat. The name of each participant is followed by the short name of their protein resource or resources in parentheses. Back row: David Landsman (Histone database), Dan Haft (TIGRFAMS), Bernard Henrissat (CAZy), Rob Finn (InterPro and Pfam), David Craik (ConoServer and CyBASE), Arnaud Chatonnet (ESTHER), Neil Rawlings (MEROPS); Middle row: Amos Bairoch (neXtProt), Gerard Manning (Kinase.com), Michael Spedding (IUPHAR), Gert Vriend (GPCRDB), Milton Saier (TCDB), Pantelis Bagos. (OMPdb); Front row: Narayanaswamy Srinivasan (KinG), Ramanathan Sowdhamini (PASS2), Alex Bateman. (Pfam & UniProt), Patsy Babbitt (SFLD), Kim Pruitt (RefSeq), Claire O’Donovan (UniProt), Gemma Holliday (MACiE) and Nozomi Nagano (EzCatDB).

Twenty-one principal investigators, each maintaining either a specialized protein bioinformatics database or a global protein resource at a large Bioinformatics centre attended this meeting. The participants at the meeting are pictured in Figure 1, and the resources they represent are listed in the figure caption. Supplementary file S1 contains a short description of each of these resources, including those not described in the body of this report. Of course there were many relevant specialist protein resources that were not included due to limited space at the meeting. According to the Oxford University Press Online Molecular Biology Database Collection (3) there are 94 database resources that focus on one or a small number of protein families (http://www.oxfordjournals.org/our_journals/nar/database/subcat/3/10). In our future activities we hope to engage as widely as possible with this larger ecosystem of specialist protein resources.

### Meeting highlights

The retreat was divided into five sessions that addressed the issues facing the resources from a variety of different perspectives.

### Session 1: Key challenges

The first session aimed to identify common challenges that faced the participants in delivering their protein resources. To help foster discussion, the session began with three short presentations by Amos Bairoch (The challenges of integrating protein-centric resources with genomic-centric resources), Bernard Henrissat (Functional predictions: The good, the bad and the ugly) and Dan Haft (Biocuration Challenges for High Dimensional Data: Derived Objects, Dark Matter and Emerging Reasoning Methods). Their presentations covered broad themes concerning the difficulties of accurate protein functional assignment, keeping genomic and protein data synchronized, missing data and the provenance of data. The ensuing discussions identified a comprehensive list of 30 challenges. An in-depth description of this session is submitted elsewhere, and we refer the reader to that publication (4).

### Session 2: Introduction to the protein resources

An important goal of the meeting was to foster communication between specialist protein resources. We found that very few of the participants had met each other face-to-face despite the often close similarities in the work they perform. The second session gave the participants an opportunity to briefly introduce their protein resources. Twenty of the participants gave 5 min lightning talks using just two slides, a task that was challenging given the richness of their resources. Some participants had a greater challenge of introducing several databases in their talks such as Pantelis Bagos from the University of Thessaly who introduced gpDB and ExTopoDB as well as OMPdb (5). However the participants rose to this challenge, and there was a real sense that common ground was established. The participants agreed that this was an important outcome for the meeting as building connections between these resources is a first step to building meaningful collaborations.

### Session 3: Best practices

The aim of this session was to identify best practices for maintaining and curating specialized resources. Four speakers were asked to present aspects of their curation, website or software tools that they thought could be adopted by others. Gert Vriend began the session by presenting his 10 rules for making a biological database. These rules had been developed through his experience in creating and running the GPCRDB (6). They are aimed at offering guidance in making a successful, long term and sustainable resource. The presentation sparked a lively discussion, and as the participants agreed and endorsed these rules, they are presented in full in Gert’s vernacular below:

1. Longevity: The one rule to rule them all. Gert asks that unless you can maintain your database for at least 10 years, then do not start.
2. Users: All databases need users and citations. To gain and keep users, you need to provide query and browsing interfaces as well as someone who answers emails.
3. Befriend *Nucleic Acids Research* and *Database* journals: The descriptions of your database are essential to inform new users. But it is also essential to target publications to the readership.
4. Collaborate: Your collaborators may offer an exit strategy in the future.
4a. Be open: Nobody is going to steal your resource.
5. Give credit: There is more than 100% to go around.
6. Automate: Too much manual intervention makes for an unsustainable database leading to premature death. You need to automate roughly 90% of everything every year.
7. No new standards: Don’t invent a new standard. Use what exists.
8. Keep it simple: Google is a model interface.
9. Visibility: Be at the right conferences and be recognizable. Use the same logo and present a poster.
10. Exit strategy: At some point you will retire. Start planning early to ensure your database continues.

David Landsman presented the Conserved Domain Database (CDD) at NCBI (7). He described the importance of two aspects of their curation activities, first, that each of the alignments for CDD families were based on structural superpositions, manually edited to improve quality, and second, that CDD families can sometimes be split according to evolutionary history to increase the functional specificities of the families. Nozami Nagano presented EzCatDB, the Enzyme Reaction Database (8). EzCatDB provides a hierarchical classification of enzyme reactions which takes particular care in curating the reaction intermediates. An Excel-based literature manager was presented which could be more widely used. Finally, Milton Saier presented the TCDB database. Over the past 20 years numerous tools have been developed with a focus on transporter proteins, including G-BLAST for annotating genomes and the SuperFamilyTree (SFT) programs which allow construction of phylogenetic trees showing protein, subfamily or family relationships based on BLAST bit scores (9). In addition, Milton stressed the usefulness of having a Scientific Advisory Board for biological databases.

### Session 4: Information flow

The aim of this session was to discuss how to improve the flow of information both to and from the large data centres such as the National Center for Biotechnology Information (NCBI) and the EMBL-European Bioinformatics Institute (EMBL-EBI).

Rob Finn’s presentation was entitled, ‘Challenges of integrating different resources into a single service and/or database’. He described the challenges faced by InterPro in integrating its 11 different protein family databases. The main message was that growth of the sequence databases puts pressure on the computational pipelines and consequently, there is continual pressure to move to faster search technologies and infrastructures. Gemma Holliday’s talk on interoperability and communication between databases introduced the large array of existing enzyme databases (see Supplementary file S1). The main challenge was that these resources operate from a variety of different perspectives such as protein centric or chemistry centric. The solution proposed was the adoption of a common language to interconnect them. The Enzyme Mechanism Ontology was presented as one option. Kim Pruitt gave the final talk in this session about information flows into NCBI (RefSeq, Gene). Kim talked about the GenBank submission pipeline and how data flowed into RefSeq. An important distinction, which applies generally to biological databases, was made between GenBank which is an archival resource, and RefSeq that is a derived database that can continually improve its records. Another important point was that RefSeq has connections with UniProt that help to reduce duplication of effort, providing a model for other curation resources. The final part of the presentation described the NCBI LinkOut system that allows external resources to have links from NCBI pages. This is a useful mechanism to help raise awareness of specialist protein resources among users.

### Session 5: Communication and funding

The final session covered communication and funding. These two issues had been raised at numerous points throughout the meeting, and the final session gave an opportunity to bring all of these threads of discussion together. This session began with three short presentations. First, Patsy Babbitt outlined some possible directions and points for discussion. Second, Michael Spedding discussed ‘IUPHAR, melding and managing complex datasets’. Michael explained the motivation and some history of the IUPHAR/BPS Guide to PHARMACOLOGY (GtoPdb) and described the considerable effort expended together with the community to define a consistent nomenclature for various protein types. The IUPHAR has over 90 committees dedicated to describing a variety of drug target families. The final presentation was by Claire O’Donovan on ‘Leveraging and sharing curation for mutual benefit’. This presentation gave an overview of communication from the perspective of the large protein resource, UniProt. The core role of UniProt curators was described followed by a description of ongoing collaborations with specialist protein resources, and Claire presented a curator wish list. These wishes included increased publication and recognition of the work of curators and improved attribution and provenance for assigning credit. Raising the profile of curators is essential for funders to recognize the need for expert curation.

It was clear that many of the specialist resources were small in terms of the number of full time employees. Most resources have at most two posts, and many had little or no grant funding, often relying on core institutional funds. It was felt that the resources were often undervalued given the high level of access and citations. There was discussion on the importance of showing the support of the community for the resources through letters of support for grant funding applications. The biological database community is international, while the grant funding landscape is extremely varied among countries. There was thought to be opportunities for transnational grant funding to support the coordination of clusters of related resources. It was concluded that grant funding or lack thereof was one of the greatest barriers to sustainability in running a specialist protein resource.

### The Specialist Protein Resource Network

A major outcome of the meeting was the creation of the Specialist Protein Resource Network (SPRN). The SPRN group aims to continue the discussions started in this retreat as well as foster future coordination and integration activities in the area of protein resources. If you are involved in running a specialist protein resource, planning to initiate one, or just interested in this topic then we invite you to join us. You can sign up for the SPRN e-mail list at this URL: https://listserver.ebi.ac.uk/mailman/listinfo/sprn.

## Supplementary Material

Supplementary Data

## Supplementary Data

*Conflict of **interest*. None declared.

## References

[1] Lombard V. Golaconda Ramulu H. Drula E. (2014) The carbohydrate-active enzymes database (CAZy) in 2013. Nucleic Acids Res., 42, D490–D495. 24270786 10.1093/nar/gkt1178 PMC3965031

[2] Saier M.H. Jr. Reddy V.S. Tamang D.G. (2014) The transporter classification database. Nucleic Acids Res., 42, D251–D258. 24225317 10.1093/nar/gkt1097 PMC3964967

[3] Galperin M.Y. Rigden D.J. Fernandez-Suarez X.M. (2015) The 2015 Nucleic Acids Research Database Issue and molecular biology database collection. Nucleic Acids Res., 43, D1–D5. 25593347 10.1093/nar/gku1241 PMC4383995

[4] Holliday G.L. Bairoch A. Bagos P.G. (2015) Key challenges for the creation and maintenance of specialist protein resources. *Proteins*., 83, 1005–1013. 25820941 10.1002/prot.24803 PMC4446195

[5] Tsirigos K.D. Bagos P.G. Hamodrakas S.J. (2011) OMPdb: a database of {beta}-barrel outer membrane proteins from Gram-negative bacteria. Nucleic Acids Res., 39, D324–D331. 20952406 10.1093/nar/gkq863 PMC3013764

[6] Isberg V. Vroling B. van der Kant R. (2014) GPCRDB: an information system for G protein-coupled receptors. Nucleic Acids Res., 42, D422–D425. 24304901 10.1093/nar/gkt1255 PMC3965068

[7] Marchler-Bauer A. Derbyshire M.K. Gonzales N.R. (2015) CDD: NCBI's conserved domain database. Nucleic Acids Res., 43, D222–D226. 25414356 10.1093/nar/gku1221 PMC4383992

[8] Nagano N. Nakayama N. Ikeda K. (2015) EzCatDB: the enzyme reaction database, 2015 update. Nucleic Acids Res., 43, D453–D458. 25324316 10.1093/nar/gku946 PMC4384017

[9] Chen J.S. Reddy V. Chen J.H. (2011) Phylogenetic characterization of transport protein superfamilies: superiority of SuperfamilyTree programs over those based on multiple alignments. J. Mol. Microbiol. Biotechnol., 21, 83–96. 22286036 10.1159/000334611 PMC3290041

