# Supplementary material for: Creating a specialist protein resource network: a meeting report for the protein bioinformatics and community resources retreat
Source: Database (Oxford). 2015 Jul 11;2015:bav063. doi: 10.1093/database/bav063 (PMC4499208; doi:10.1093/database/bav063)
Supplement: Supplementary Data [file supp_2015_bav063_index.html]

Supplementary Data 

# Creating a specialist protein resource network: a meeting report for the protein bioinformatics and community resources retreat

## Supplementary Data

files

- Supplementary Data - zip file
