# Supplementary material for: Creating a specialist protein resource network: a meeting report for the protein bioinformatics and community resources retreat
Source: Database (Oxford). 2015 Jul 11;2015:bav063. doi: 10.1093/database/bav063 (PMC4499208; doi:10.1093/database/bav063)
Supplement: Supplementary Data [file supp_bav063_suppl_data.zip › FileS1 - Database editorial-AV June22.docx]

**Supplementary File 1**

Creating a Specialist Protein Resource Network: A meeting report for the Protein Bioinformatics and Community Resources Retreat.

Organizing data in a database

This meeting review will address the questions and problems facing database managers and lend insight as to why experimentalists might wish to publish their data in a database rather than a website or traditional journal articles. We hope it will provide inspiration for experimentalists to start specialized database resources, especially when the reams of data now obtainable are difficult or impossible to present in traditional sources. The basis for the discussion presented below resulted from a policy meeting, sponsored by the Wellcome Trust in Hinxton, England on August 11-12, 2014. Twenty one principal investigators, each maintaining a specialized protein bioinformatics database or doing research pertaining to such a resource, attended this meeting which was divided into five sections, (1) key challenges, (2) database introductions, (3) best practices for maintenance and curation, (4) information flow to and from large data centers, and (5) communication and funding. A list of the specialized protein databases represented is presented below (see Table S1). A concluding section summarizes the overall sentiments that resulted from the meeting in Hinxton.

{Placeholder of Tables S1, S2}

The Wellcome Trust SPR Network meeting brought together researchers who maintain ‘niche’ online databases that are devoted to a specific type of protein (as defined by enzymatic, functional, or structural characteristics), as well as managers of large protein resources (including Pfam ([1](#_ENREF_1)), RefSeq ([2](#_ENREF_2)), Swiss-Prot ([3](#_ENREF_3)) and UniProt ([4](#_ENREF_4))) that aim to represent all proteins organized by organism, by sequenced genome, by availability of experimental data, or global definitions of protein domains. These large data centers use several approaches to maintain data content including computational analysis, collaboration, data integration from multiple sources, and curation. All of the participating resources are supported by biocuration to ensure accuracy and completeness of the data presented by each large or small protein database resource. A common concern shared by all participants is the burden of curation as it is often challenging to extract information from publications when the articles do not provide specific information about an organism (and breed/cultivar/ecotype/strain) studied, or the accession plus version number (or gi) of the sequence(s) analyzed. Accordingly, expanding collaborations to correct errors across multiple databases and to share expert knowledge was identified as a high-value activity that would benefit all of the protein resources and users of those resources.

**CAZy: the Carbohydrate-active enzymes database**

The CAZy database ([www.cazy.org](http://www.cazy.org)) describes the families of enzymes that break down, modify, or build glycosidic bonds. CAZy has been recently described ([5](#_ENREF_5),[6](#_ENREF_6)) in terms of contents so here we will cover only some nuts and bolts of this operation. The sequence family classification of glycoside hydrolases that eventually became the CAZy database was published in 1991, before any cellular genome had been sequenced ([7](#_ENREF_7)). This classification was extended to other categories of carbohydrate-active enzymes such as the glycosyltransferases ([8](#_ENREF_8)) and released as an online website in September 1998, based on HTML pages derived from tab-delimited tables. In 1999 the data were imported into an SQL-database to facilitate day-to-day management.

The daily inclusion of new sequences to existing families, the occasional creation of a novel family (always based on published experimental evidence), the survey of the literature to find published activities and the analysis of PDB entries is performed by two scientists (curators) and two engineers (database developments and development of tools to improve curation throughput). Despite the rapid increase in sequence data, each and every sequence that appears in CAZy is still reviewed by a curator, unless the new sequence aligns with no gap and with >50% sequence identity with a sequence already classified. The human curation step, which includes error corrections at the demand of users, and the assignment of EC numbers only to experimentally characterized enzymes with no annotation transfer by sequence similarity, has made CAZy a reference resource in the glycosciences.

A database of enzyme families needs to be completed by an encyclopedic resource to provide researchers an accurate survey of the knowledge within a family. This realization was the main motivation behind the development of the complementary CAZypedia website (<http://www.cazypedia.org>), the logical extension of the CAZy database. CAZypedia is headed by Professor Harry Brumer of the University of British Columbia, assisted by a board of senior curators who solicit responsible curators and specialist contributors to write summaries on each family in their own real names, thereby involving the community of glycoscientists at large. Thus, this community adheres to and follows the naming conventions of the CAZy classification system. As a consequence, it is frequent that those who discover a new family of CAZymes approach the CAZy database ahead of publication, in order to request a CAZy family number to use at publication time. Similarly, when a new activity in an existing family is discovered, we sometimes receive note from the authors of the work, in order to complete the functional information of CAZy.

The future of CAZy depends on its ability to provide a useful service and analytical system, beyond being a hierarchical archive of family names and sequences. We thus develop new facets to our database such as the capture and display of the carbohydrate ligand information in three-dimensional structures of carbohydrate-active enzymes, and the capture and organization of functional information such as substrate specificity. The latter has particular significance when attempting to predict the function of gene products for genomic annotation. Despite our efforts, the experimental information that is presented in CAZy is necessarily incomplete. Researchers can help by providing CAZy with substrate/product data they have published that have escaped our attention. Because modern biochemistry is progressively generating very large data sets with activities reported for dozens (and soon thousands) of enzymes in one single paper, it would be a great advance if researchers would deposit their data as supplementary material in a tabular format that would include sequence database accession for each characterized enzyme, the substrates used and the products detected. If scientific journals would make such simple and minimal data deposition compulsory, it would allow a more complete and reliable data capture into our database to everyone's benefit. We believe that such a practice would facilitate functional data capture well beyond CAZy.

**ConoServer**

Cone snail venoms are a large pool of potentially several hundreds of thousands of active peptides exquisitely selective for receptors and transporters of the nervous systems with applications as neurological probes and drug leads ([9](#_ENREF_9),[10](#_ENREF_10)). The large inter- and intra-species variability of these venoms ([11](#_ENREF_11)) has been analyzed in genetic ([12-14](#_ENREF_12)) and ecological ([15](#_ENREF_15),[16](#_ENREF_16)) studies of their evolution. As of December 2014, the ConoServer database ([17](#_ENREF_17)) catalogs more than 2000 conopeptides and helps to systematise the three classification schemes that broadly describe conopeptide evolution, three-dimensional folds and molecular targets ([18](#_ENREF_18)).

ConoServer ([www.conoserver.org](http://www.conoserver.org)) was designed to be managed (and its entries annotated) with limited human resources, *i.e.* one person. This goal was achieved by implementing an additional layer, coined pseudo-tables that links the data stored in a relational MySQL database to a PHP Web interface. ConoServer uses a set of XML files that define pseudo-tables of proteins, nucleic acids, three-dimensional structures and literature references, corresponding to all sections of the Web interface. Each pseudo-table field is populated by a PHP module that processes data provided by the MySQL server. The pseudo-table XML files describe SQL access to the MySQL database and inform the PHP Web interface how the pseudo-tables could be queried and how and where each of their fields should be displayed. Addition or modification of queried and displayed data therefore only requires limited editing of the XML files, which are used to automatically generate the query forms and individual toxin pages. Modification of the structure of the MySQL database is also transparent to the Web interface as all accessible data originate from the pseudo-table layer.

The primary data underpinning ConoServer are sourced from GenBank ([19](#_ENREF_19)), UniProt-KB ([4](#_ENREF_4)) and the wwPDB ([20](#_ENREF_20)) as well as from the literature or through direct submissions by authors. We discourage direct submissions to ConoServer as we prefer the sequence and structural data to be sent to main repositories from which they are automatically fetched every month. Newly retrieved sequences are first annotated by several scripts that enter data in the MySQL database, and these data are then reviewed and modified manually through a Web annotation interface before being released. A number of conopeptide sequences are available only in tables, figures or supplementary information of peer-reviewed articles, and they are entered manually through a Web interface. Conopeptide protein and nucleic acid precursor sequences are annotated by identifying their sequence features as well as classifying them according to the three standardized classification schemes. ConoPrec ([17](#_ENREF_17)) is a Web tool that allows the use of this annotation procedure, helping users analyze sequences using ConoServer standards before publishing them, hence simplifying their later entry into ConoServer. The consistency of ConoServer data is regularly checked through a range of tests implemented in scripts that generate text reports.

**CyBase**

Ribosomally synthesized, head-to-tail cyclic backbone proteins have been discovered in all kingdoms of life ([21](#_ENREF_21),[22](#_ENREF_22)). Backbone cyclization renders proteins impervious to exoproteases and results in a dramatic improvement of stability against enzymatic degradation as well as against thermal or chaotrope denaturation ([23](#_ENREF_23)). This high stability of cyclic peptides and proteins has attracted considerable interest from drug designers to stabilize bioactive peptide epitopes ([24](#_ENREF_24)). CyBase ([www.cybase.org.au](http://www.cybase.org.au)) is a database that provides access to information on gene-encoded, backbone, cyclic proteins ([25](#_ENREF_25)). As of December 2014, CyBase contains information on *ca.* 420 naturally occurring circular proteins and *ca.* 160 synthetic circular proteins. These proteins have been classified into nine major classes, the largest of which is the cyclotide class, with 282 entries. The implementation and annotation strategies in CyBase are very similar to those described for ConoServer. Circular proteins pose specific bioinformatics challenges because most algorithms and tools are designed for linear sequences. CyBase search algorithms have been adapted to handle circular proteins, the most frequently used tools being sequence alignment and mass-spectrometry finger print searches ([25](#_ENREF_25)). A distinctive feature of CyBase is its in-depth textual description of biological assays and physicochemical characterizations of each circular protein.

**ESTHER**

The ESTHER Database (ESTerases and alpha/beta-Hydrolase Enzymes and Relatives) is dedicated to the analysis of proteins belonging to the superfamily of alpha/beta-hydrolases ([www.bioweb.supagro.inra.fr/esther](http://www.bioweb.supagro.inra.fr/esther)). Alpha/beta hydrolases constitute one of the largest and most diverse protein superfamily characterized by a single structural fold. ([26](#_ENREF_26)). Subfamilies are built according to an HMM profile ([27](#_ENREF_27)). Members of the superfamily play fundamental roles in virtually all physiological processes and are targeted by drugs aimed at treating diseases such as diabetes, obesity, and neurodegenerative disorders. Despite what their generic names would suggest, many of these proteins are not enzymes as some have lost all residues necessary to constitute an active site ([28](#_ENREF_28),[29](#_ENREF_29)). The functions of few representatives of this last group are known: intracellular receptors of small molecules, endo cellular membrane trafficking agents, inter-actors with partners at cell-cell interfaces, and hormone precursors. The database was launched in 1994 with a Gopher server and quickly moved to WWW ([30](#_ENREF_30)). The underlying system of the database is the venerable ACeDB system. ACeDB is a genome database system developed in 1989 for the *C. elegans* genome project. It is still the kernel of the Wormbase database and server. The initial interface to the web is based on CGI scripts AcePerl. As a database engine, ACeDB compares favorably with MySQL or even commercial database systems. Complex queries can be easily built ([31](#_ENREF_31)). Examples of dedicated searches have been published ([32](#_ENREF_32)). The database also contains small molecules interacting with esterases as substrates, inhibitors or activators and associated kinetic data ([33](#_ENREF_33)).

**Enzyme Catalytic Mechanisms Database (EzCatDB)**

EzCatDB (http://ezcatdb.cbrc.jp/EzCatDB/) was first released in 2004 in order to provide a manual classification of enzyme reactions in terms of enzyme active-site structures and their catalytic mechanisms. It provides the hierarchical reaction classification, RLCP, based on literature information ([34](#_ENREF_34),[35](#_ENREF_35)) and distinct from the Enzyme Commission (E.C.) (or NC-IUBMB; <http://www.chem.qmul.ac.uk/iubmb/enzyme/>) ([36-38](#_ENREF_36)). As the database management system, PostgreSQL has been adopted for the development of the core part of EzCatDB, so that a search for enzyme data in EzCatDB can be specified in various ways ([34](#_ENREF_34)).

For EzCatDB, the information from various resources (Protein Data Bank (PDB) ([39](#_ENREF_39)), UniProtKB (74), CATH ([40](#_ENREF_40)) and KEGG ([41](#_ENREF_41))) including the literature for each enzyme entry are analyzed and put together into pre-defined Excel formats by our curator, and after the final check, the enzyme entry is uploaded into the EzCatDB. Accordingly, all processes involving manual annotation are time-consuming and challenging, due to the large amount of data and our language handicap for the literature. Particularly, the extraction and analyses of literature information are the most time-consuming. However, they are extremely important for the EzCatDB database. The section concerning MEROPS also mentioned that a full-text search is not always possible through the PubMed interface ([42](#_ENREF_42)), since the essential data are in figures that cannot be searched electronically. The scheme for extraction of literature information for EzCatDB is described below.

Although EndNote or other software might be used to manage the literature information, an Excel-based literature management system has been prepared for the EzCatDB literature information. After a PubMed search ([42](#_ENREF_42)), the literature text data is converted into an Excel-formatted literature list using a Visual Basic program. Then, it is checked to determine if each literature element overlaps with the previously retrieved literature, if a corresponding PDF file is available, or if it should be copy-ordered for our library. To find previously retrieved literature, another Excel-based system to search with PubMed IDs has been developed. For the literature, whose PDF file is not available through online journals, a photocopy must be ordered for our library. An advantage of this Excel-based literature manager is that the web-based library order-form can be filled in automatically. Moreover, the Excel-based literature manager can be customized to any system change relatively easily. If the PDF files are available, searches with keywords such as “active site”, “general acid” and “nucleophil” (here, “nucleophil” can cover both “nucleophile” and “nucleophilic”), and then batch processing of highlighting the identified keywords are performed, using Acrobat software. Consequently, it is much easier for us to find appropriate paragraphs or even figure legends of enzyme reaction mechanisms with the PDF files. More recently, a scanning system, ScanSap SV600 (Fujitsu), has been introduced to convert paper-based literature into PDF files, so that extractions of literature information can be carried out more efficiently. The obtained literature information is further analyzed to annotate active-site residues, to classify the enzyme reaction types, and to create ligand data, for which KEGG compound data are not available ([35](#_ENREF_35)).

Although only limited amount of enzyme data are available, 300 more entries are still being prepared, which is challenging with limited manpower and funding. Only one scientist (Nozomi Nagano) with a technical staff as annotators is now working to curate EzCatDB. Additionally, a system engineer is working remotely outside our institute, together with supporting engineers and collaborating scientists, for the development and maintenance of the EzCatDB system. For long-standing and more efficient database curation, communications with other enzyme-related databases must be instigated.

**G Protein-coupled receptor database (GPCRdb)**

G protein-coupled receptors (GPCRs) make up the largest membrane protein family in certain eukaryotes ([43](#_ENREF_43),[44](#_ENREF_44)), and constitute the targets of roughly 30% of marketed drugs ([45](#_ENREF_45),[46](#_ENREF_46)). The GPCR database, GPCRdb ([gpcrdb.org](http://www.gpcrdb.org)), was started by Gert Vriend, Ad IJzerman, Bob Bywater and Friedrich Rippmann in 1993. In 2013, GPCRdb was transferred to the Gloriam group at the University of Copenhagen, backed up by an EU COST GPCR Action ‘GLISTEN’. In this process, the database has transformed from being mainly a bioinformatician’s resource to target a multidisciplinary audience with the release of new diagrams and tools; as well as cross-referring with major complementary databases ([47](#_ENREF_47)).

GPCRdb contains the largest collections of receptor *in vitro* mutants, obtained during years of curation. As an open archive, GPCRdb also allows researchers to contribute mutagenesis data to increase its dissemination. The mutants are often displayed within receptor residue diagrams, which can be custom-coloured and downloaded for use in publications/presentations. GPCRdb also maintains a curated reference collection of all GPCR crystal structures, which have increased exponentially in numbers due to recent technological breakthroughs ([48](#_ENREF_48)). Additional tools allow, for example, superposing on the overall structure or sub-site residues that constitute a common ligand-binding pockets.

Correct protein sequence alignments are a pre-requisite for many studies on receptor mutants, ligand-binding residues and functional structural microdomains. Consequently, GPCRdb has released (crystal) structure-based sequence alignments that are unique in that they take into account structural distortions so that spatially equivalent residues aligned in a structural comparison are also the ones aligned in sequence ([49](#_ENREF_49)). This includes cross-class sequence alignments, allowing for studies of conserved motifs of the GPCR machinery. GPCRdb alignments feature residue conservation statistics for the 20 individual amino acid types as well as property groups, such as hydrophobicity, charge and size.

GPCRdb is in the process of migrating to modern web technologies that are independent of third party web browser extensions. The new interface uses HTML5 technologies to provide a responsive user experience. Scalable vector graphics (SVG) are used to generate downloadable, interactive diagrams. GPCRdb offers SOAP web services for programmatic access, and is working towards making more content dynamically accessible.

Long-term sustainability is a major consideration. To engage more developers, GPCRdb will be made available open source, and data deposition will be facilitated by standardised web forms and Excel sheets (like those currently available for mutant data). Development is funded for the five coming years, but we continuously secure new grants since GPCRdb is an integral part of many interdisciplinary projects, both as a research tool and as a dissemination instrument.

In conclusion, GPCRdb aims to enable research by providing easy access to key experimental and derived data, visualise relationships and to provide easy-access analytical tools. The GPCR field and GPCRdb have experienced fundamental changes during the past twenty years, and the database intends to grow with and to inspire further scientific progress in the future.

**GtoPdb - IUPHAR/BPS Guide to PHARMACOLOGY**

The IUPHAR/BPS Guide to PHARMACOLOGY database (GtoPdb, ([50](#_ENREF_50))) was developed jointly by the International Union of Basic and Clinical Pharmacology (IUPHAR) and the British Pharmacological Society (BPS) to provide access to high quality information on pharmacological targets. GtoPdb (<http://www.guidetopharmacology.org/>) builds upon two previously separate but complementary resources, the IUPHAR database (IUPHAR-DB, ([51](#_ENREF_51))) and the Guide to Receptors and Channels (GRAC) publications (e.g., ([52](#_ENREF_52))).

The GtoPdb model is to have curators for particular protein target classes, linked to expert subcommittees who review the information. The IUPHAR nomenclature committee, NC-IUPHAR, with its 90 subcommittees representing >650 scientists, coordinates the development of the database. NC-IUPHAR publishes nomenclature recommendations and reviews (H-Index 72) on various topics in pharmacological journals and via the database.

GtoPdb aims to (i) provide access to data on all known biological targets; (ii) make recommendations on ligands for use in characterising those targets; (iii) provide an entry point into the pharmacological literature; (iv) provide access to educational resources with training in the principles of basic and clinical pharmacology; (v) foster innovative drug discovery.

It now includes >2700 established or potential drug targets and related proteins (G protein-coupled and other types of receptors, ion channels, kinases, proteases, transporters and other targets), with the key ligands which are either marketed drugs or drugs in development, or the best available experimental tool compounds.

Thus GtoPdb does not have the scope of capture that ChEMBL ([53](#_ENREF_53)) does; however, it complements large scale approaches in being a focused, expert-curated database of carefully selected ligands and targets. It provides background information and commentary to add context. The data are extensively cross-referenced to other protein and gene databases (e.g. UniProtKB ([4](#_ENREF_4),[54](#_ENREF_54)), Ensembl ([55](#_ENREF_55)), KEGG ([41](#_ENREF_41))), chemistry resources (e.g. PubChem ([56](#_ENREF_56)), DrugBank ([57](#_ENREF_57)), ChEMBL), and to primary literature in PubMed ([42](#_ENREF_42)). Subcommittees update their database pages annually with public database releases every 3-4 months.

There is also a ‘Concise Guide to PHARMACOLOGY’ ([58](#_ENREF_58)) published biennially in the *British Journal of Pharmacology*, which is a snapshot created from the target family summaries in GtoPdb, and serves as a quick desktop reference guide and citable record.

GtoPdb records affinity values mapping bioactive chemical structures to proteins. However, there are so many variables associated with receptor affinity that it becomes problematic for data-mining, and expert pharmacologist subcommittees are essential ([59](#_ENREF_59)). Variables such as ncRNAs (in progress, with HGNC), epigenetics ([60](#_ENREF_60)), alternative splicing ([61](#_ENREF_61)), allostery ([62](#_ENREF_62)), and immune interactions (in progress), contribute significantly to disease processes ([59](#_ENREF_59)) and create major difficulties for drug discovery. It is beyond the resources of most groups to take all of the issues into account and GtoPdb provides access to the current status of knowledge. NC-IUPHAR enables experts to establish guidelines for the field, define the difficulties and opportunities, and produce highly cited publications.

The resource is financed by a Wellcome Trust grant (099156/Z/12/Z), and by IUPHAR, BPS and pharmaceutical industry contributions. The key role of expert subcommittees is a major advantage ensuring validated data, and their time and effort is much appreciated.

**The Histone Database**

The Histone Database (<http://research.nhgri.nih.gov/histones/>) was established in 1996 ([63](#_ENREF_63)) as a result of research on the histone fold in DNA binding proteins ([64](#_ENREF_64)). The database is updated on an irregular basis, and the updating process is frequently used as a tool to instruct undergraduate, doctoral, and/or postdoctoral students entering computational biology/bioinformatics on database design and sequence analysis. Over the years we have used several different tools to identify histone proteins in the sequence databases including the current versions at the time of PSIBLAST ([65](#_ENREF_65)) and HMMER ([66](#_ENREF_66)). After identification using these tools, we inspect the sequence alignments and check for incorrect entries in the protein databases. Most of these errors comprise the inappropriate assignment of start codons. Since histone proteins are highly conserved, and the histone fold is well described and annotated, it is quite easy to identify histone proteins which are incorrectly annotated in the public databases. Alignments of each family of histone (H1, H2A, H2B, H3, and H4) are available for download, and there is also a query page to extract only histone sequences of interest. We have used these alignments to help us identify and update the histone fold proteins ([64](#_ENREF_64)) as well as the recent addition of the family of archaeal histones ([67](#_ENREF_67)). These latter protein collections are listed in the Histone Database as separate families. An interesting example of the histone fold is in Son-of-Sevenless (Sos) which dimerizes with another histone fold in the same polypeptide ([68](#_ENREF_68)). We had previously predicted both these regions as histone folds. The Histone Database also contains lists of histone 3D structures extracted from the Protein Data Bank (PDB) ([69](#_ENREF_69)), mostly in the form of nucleosome structures determined by X-ray crystallography.

Future challenges include updating the Histone Database with new sequences in the public databases and the subdivision of each histone family into the various sub-families or variants (*e.g.* the centromeric histones (CENP-A and CSE4), histones H3.3, H2A.B, H2A.Z, H2B.Z, macroH2A ([70](#_ENREF_70)) and possibly also the different histone H1 variants).

**Kinase.com**

[www.Kinase.com](http://www.Kinase.com) explores the functions and evolution of protein kinases, which are key controllers of most biochemical pathways and important in health and diseases ([71](#_ENREF_71)). It focuses on the kinome, the complement of protein kinases in any sequenced genome. The website includes an interactive database, KinBase, which holds the information on over 7000 protein kinase genes found in the genomes of human and 14 other sequenced genomes ([72-77](#_ENREF_72)). These protein kinases are classified into a hierarchy of 10 groups, 287 families and 356 subfamilies. Users can search KinBase by a variety of different gene names and accessions, accessory domains, or according to the classification. In addition, the website provides BLAST services to search kinases by sequence similarity. Each protein kinase has its own page that contains its classification, sequence, annotation from external sources, domain combination graph, and particularly, a comparison to in-house HMM profiles of kinase groups, families and subfamilies. Each protein kinase class (group, family and subfamily) has its own page that contains a sequence alignment, HMM profile and phylogenetic tree of protein sequences and kinase domains. In addition to KinBase, Kinase.com has a public wiki system, WiKinome, focusing on kinase evolution and function. The ultimate goal is a wiki page for every kinase family and subfamily.

Kinase.com was created in 1999 by Sucha Sudarsanam of the drug discovery company Sugen to support the publication of Sugen’s analysis of the protein kinases of *Caenorhabditis elegans* ([78-80](#_ENREF_78)) and *Saccharomyces cerevisiae* ([81](#_ENREF_81)). The database KinBase was developed in 2002 by Gerard Manning and Glen Charydczak to support further work on protein kinases of humans ([71](#_ENREF_71)) and fruit flies ([82](#_ENREF_82)). It was implemented in MySQL database format with a Common Gateway Interface (CGI) front end implemented in Perl. Since 2004, Kinase.com has been maintained by Gerard Manning’s research group. It was hosted by the Salk Institute from 2004 to 2013 and is currently hosted with the financial support from Genentech. The website has recently been revamped by using modern web development technologies including Model–view–controller web framework, HTML5, CSS5 and JavaScript.

The data in kinase.com, including classification, annotation and sequences, are represented as relational models and stored in multiple MySQL tables. Using the MySQL database in Kinase.com, it is easy to: i) carry out complex queries quickly, ii) watch for data integrity, particularly during maintenance and curation, and iii) migrate to a new web server. Although the MySQL database has many benefits, it is not a simple task to retrieve the data from MySQL and present them in dynamic pages. To leverage the MySQL database, we use Model–view–controller web framework Django for front end development. Django has an object-relational mapper (ORM) which facilitates exchange between the MySQL database and Python objects. Django also has a template system to present objects in any text-based format, such as HTML, CSV and SVG.

The key challenge is to update the database with high-quality data. Although over 6000 eukaryotic genomes have been sequenced, Kinase.com includes only the kinomes of 15 genomes. We have endeavored to automatically identify and catalogue protein kinases of all sequenced genomes, but we have not achieved the same quality as for the 15 kinomes in the aspects of gene model and/or classification. Currently, two researchers maintain Kinase.com on a part-time basis (Mark Jinan Chen and Gerard Manning). Without the manual curation from other researchers, it is difficult to provide regular and frequent updates of high quality kinomes.

**KinG**

Database of Kinases encoded in Genomes (KinG) is a resource on Ser/Thr/Tyr kinases ([83](#_ENREF_83)). Currently the full complement of protein kinases in various completely sequenced genomes is hosted in the Garuda India network at http://megha.garudaindia.in/king/ which provides a detailed listing of the Ser/Thr/Tyr and atypical protein kinases in various organisms, accompanied by other features such as protein kinase subfamily classification and domain organization. In the very first version of KinG published in 2004 ([83](#_ENREF_83)), kinases from only 40 organisms were documented. KinG is updated every year. Kinases are highly represented, especially in eukaryotes. Further, as the number of completely sequenced genomes is mounting rapidly, with every update, the number of kinases that need to be handled also grows. In the current version of KinG, 12200 genomic datasets have been screened resulting in the identification and classification of 131921 kinases. Hanks and Hunter’s classification scheme ([84](#_ENREF_84)), tailored with a multiple position-specific scoring matrices (PSSM) approach ([85](#_ENREF_85)), has been followed in KinG to classify kinases into subfamilies. A challenge here is the inconsistency between the subfamily classification, which is based purely on the sequences of catalytic kinase domains, and the domain combinations of the multi-domain kinases. Our recent analyses ([86](#_ENREF_86),[87](#_ENREF_87)) enabled us to recognize the occurrence of hybrid kinases which are characterized by a subfamily of kinase, recognized based on the sequence of the catalytic domains alone, and characterized into another subfamily of kinases based on consideration of the domain architecture. This complex situation does not permit us to classify such a kinase into either one of the subfamilies, and therefore we propose that these are classified as hybrid kinases with characteristics of two different kinase subfamilies.

Another twist in the evolution of multi-domain kinases which causes difficulties in classification is the occurrence of rogue kinases ([86](#_ENREF_86),[87](#_ENREF_87)). Rogue kinases are characterized by association of the kinase catalytic domain with a specific sub-family of kinases, but the co-occurring domains are not characteristic of any subfamily of kinases. Therefore, such kinases display features which are deviant from the classical members of the subfamily.

We feel that the design of the proposed classification scheme of kinases is facilitated by the analysis of the emergent kinome data. The data and the classification scheme feed each other. As we constantly improve the classification scheme ([88-90](#_ENREF_88)) we continue to update the KinG database.

**The MACiE Database**

MACiE, which stands for Mechanism, Annotation and Classification in Enzymes (<http://www.ebi.ac.uk/thornton-srv/databases/MACiE>), is a database of enzyme reaction mechanisms ([91](#_ENREF_91)). MACiE collates and stores information on enzymes, their overall chemical transformations, reaction mechanisms, cofactors and catalytic residues. Every entry in MACiE is represented by at least one crystal structure in the wwPDB and a well-defined mechanism from the primary literature. The data in MACiE can be viewed as a conceptual hierarchy, which grows from the fact that an enzyme can be clearly defined by its components (Fig. S1).

{Placeholder Fig. S1}

This hierarchy allows us to define an enzyme, at its simplest, as a biopolymer that has a primary amino acid sequence and catalyzes an overall chemical transformation (this definition doesn’t include ribozymes, nucleic acid based ‘enzymes’). An overall chemical transformation *must* be made up of at least one substrate and one product, and it will have a mechanism, although the mechanism may not always be known in detail. The fact that these data can be represented in a hierarchical layout shows that they are relational and can be described in a relational database. MACiE uses MySQL. This level of relational information allows for complex queries to be performed quickly. For example, the enzymes in MACiE that utilize hydrogen peroxide as a substrate can be found as indicated in Fig. S2. Many similar hierarchical searches are already implemented on the MACiE site ([92](#_ENREF_92)).

{Placeholder Fig. S2}

By utilizing the relationships shown in Fig S1, it is simple to traverse from one datum to another, related only by the fact that it is also one of the components needed to define an enzyme. An example might be: “Now that I know all the entries with hydrogen peroxide as a substrate, what cofactors are involved?” The answers are that M0248 and M0250 both utilize heme, M0014 uses vanadate and M0239 is a so-called cofactor-free chloroperoxidase. One can also ask: “Of all the enzymes that utilize cofactor X, what are the overall reactions involved?” Are there any amino acyl residues (e.g. singletons, dyads or triads) shared by all or many of them? Such questions are much more challenging to ask without a relational data structure. Of course, to be able to ask such questions, the underlying data need to be present, and so MACiE contains a large amount of metadata that can be associated with enzymes and their reaction mechanisms. The key data include (1) the detailed functions of each catalytic amino acyl residues in the catalytic site, (2) the presence of catalytic dyads or triads, (3) the mechanistic description of each reaction step, (4) the bond changes, (5) reactive centers, cofactors and their functions, and (6) details to link out to external databases, such as the EC number, the UniProtKB identifiers, CATH, PDB codes, to name a few.

A final key component of the MACiE data structure is the use of a strictly controlled vocabulary and extensive error checking at the point of entry. We check to see if the protein is already present in MACiE and whether the amino acyl residues annotated are present in the crystal structure. Exceptions are made when the catalytic unit, the smallest unit required for catalysis to occur, does not also represent the asymmetric unit in the PDB file. We also check if annotations match the hierarchy, e.g., when a residue is annotated as a reactant but has not got a reactant function annotated. Such checks and controls allow us to minimize human error in assigning function and mechanism to entries, which is always possible with manual biocuration.

**MEROPS**

The MEROPS database (<http://merops.sanger.ac.uk>) provides a classification and nomenclature for proteolytic enzymes and the protein and small molecule inhibitors that affect their enzymatic activity ([93](#_ENREF_93)). Because enzymes such as peptidases that act on biopolymers are difficult to classify by reaction catalyzed alone (the criteria used by *Enzyme Nomenclature* ([36-38](#_ENREF_36))), the decision was taken to create a specialized database that would take into account evolutionary relationships as well as substrate specificity. The database was launched in 1996 and now includes over 400,000 sequences of peptidases. There is a hierarchical structure: sequences that share a similar protein fold are organized into a clan; sequences that share statistically significant similarity in the peptidase domain identified by BlastP or HMMER searches ([65](#_ENREF_65),[66](#_ENREF_66)), are organized into families; and members of a family are clustered into a “protein species”, which represents the same enzyme from different species of organisms, based on similar known (or predicted) specificities, a similar domain architecture and clustering together on a phylogenetic tree ([94](#_ENREF_94)). There is a unique identifier for each of the 61 clans, 251 families and 4,236 protein species identifiers. In comparison, there are only 377 peptidases included in *Enzyme Nomenclature*. The data collection has expanded to include over 28,000 peptidase inhibitors derived from gene products, and over 1,200 small molecule inhibitors. Knowing where a peptidase cleaves its substrates is important for determining the specificity of the peptidase, and a reasonably large collection of substrate cleavages can be useful for predicting where cleavage might occur in other as yet untested substrates ([95](#_ENREF_95),[96](#_ENREF_96)). The MEROPS substrate cleavage collection was set-up in 1998, and has grown to include more than 60,000 known cleavages in proteins, peptides and synthetic substrates ([97](#_ENREF_97)). Peptides and protein substrates are cross-referenced to UniProt ([54](#_ENREF_54)) and cleavage positions are numbered according to the corresponding UniProt entry. The MEROPS database also includes a collection of over 6,000 peptidase-inhibitor interactions ([98](#_ENREF_98),[99](#_ENREF_99)). Both of these data collections are unique. We cross-reference over 53,000 publications and collaborate with several other protein sequence databases including UniProt ([54](#_ENREF_54)), Pfam ([1](#_ENREF_1)) and InterPro ([100](#_ENREF_100)). Data are stored in a MySQL database and the tables are provided as SQL or text files on our FTP site (ftp.sanger.ac.uk/pub/MEROPS).

Like other protein-specific databases, we are aware of errors in the primary data that trickle through to other databases. It is almost impossible to correct errors in the primary sequence databases without the consent of the original depositors. We would ask genome annotators to be aware that enzymes, and especially peptidases, must have the full complement of active site residues to be functional, and that a coding sequence lacking any one of these should not be annotated as an active enzyme. We provide the MEROPS batch Blast service whereby a user can upload a library of sequences (in FastA format, which can be an entire proteome) and receive by E-mail a list of peptidase homologues, predictions of active site residues and indication where these are missing ([101](#_ENREF_101)). Careful scrutiny of this list can help avoid some of the common mistakes made by genome annotators.

Developing new features, maintaining the classification and filing new homologous sequences is achievable by the small team that runs the database (one full time employee, and one part time, unsalaried curator), but ensuring that the other data collections and textual summaries are complete and up-to-date is beyond what the team can do. It would help the curators and make the database more complete and accurate if members of the scientific community could provide results from their own publications to fill in the missing data and correct the errors.

Researchers can help by providing MEROPS with substrate cleavage data they have themselves identified. With more than 4,000 different peptidases recognized in MEROPS, it quickly becomes clear that the number of known cleavages per peptidase is actually quite small, and in reality some peptidases are over-represented (there are over 14,000 cleavages by trypsin, for example). More than half of the distinct peptidases have no cleavages at all. Finding substrate cleavages in publications is challenging, because such data are rarely included in the abstract. This means a full-text search is required, which is often not possible through the PubMed interface because the full text is available only to the journal’s subscribers. In many cases the essential data are in figures which are not searchable electronically or the data are present in supplementary material that the journal may no longer presents on its website.

A researcher could provide a summary description of the peptidase on which he or she works. We introduced a community annotation project in 2013 ([93](#_ENREF_93)). Providing a textual summary describing each peptidase and its biological roles requires more curator time than is currently available, and the curators can only (just) maintain the summaries for each family. Initially, known experts in the field were invited to contribute, but fewer than a hundred of these responded. Writing a summary is an ideal project for a PhD student working on a particular peptidase who has to prepare an introduction for his or her thesis, and we would encourage supervisors to contact us with a recommendation for a contributor. Each summary is highly structured to ensure consistency and can be completed through an on-line form. This is not a wiki, and the contributor remains in charge of his or her peptidase summary until he or she wishes to relinquish it. The summary is carefully checked by the MEROPS curators and the contributor is fully acknowledged on the website.

The benefits for a researcher who contributes to a biological database are: to see their contribution acknowledged, to see his or her publications given prominence, and to improve the data collections and correct the many errors, which in turn helps other researchers who utilize the data.

**neXtProt**

neXtProt (http://[www.nextprot.org](http://www.nextprot.org)) is a web-based knowledge resource on human proteins ([102](#_ENREF_102)). It integrates information originating from UniProtKB/Swiss-Prot with a wealth of other data originating from repositories and databases that store the results of high-throughput experiments in the field of proteomics, transcriptomics and genomics.

There are a number of challenges to maintaining a resource such as neXtProt. The first lies in the selection and quality grading of the information that is integrated. One of the missions of the neXtProt biocuration team is to grade experimental results into three categories: bronze (>5% error rate), silver (from 1 to 5% error rate) and gold (less than 1% error rate). Bronze data are not integrated into neXtProt. The quality grading of experimental results is generally not very easy to achieve as it is often the case that papers or data submission to repositories do not provide the necessary criteria to objectively assess the quality of the experimental setup and results. Ideally, these assessments should be revisited periodically when techniques change and are superseded by better, more accurate methods.

Another major challenge for a resource such as neXtProt that attempts to integrate a wide variety of information originating from very heterogeneous resources is the constant need to change and update the information provided in the database. neXtProt strives to follow a monthly release schedule, but this is sometimes disturbed by important changes in at least one of the integrated resources. Format changes are particularly disruptive.

Finally the integration of resources that do not share the same standardization resources is fraught with problems. It is generally necessary to produce and maintain mapping tables between different ontologies or controlled vocabularies. This problem is specifically acute concerning human diseases: there are more than 10 ontologies that are used by the medical and life science communities.

**OMPdb**

OMPdb ([103](#_ENREF_103)), available at <http://www.ompdb.org>, is a publicly available database that contains β-barrel outer membrane proteins from Gram negative bacteria. It was first launched in 2011 containing ~70,000 protein entries. whereas currently it includes >500,000 featured entries. Each family is represented by a characteristic profile Hidden Markov Model (pHMM). Most of the families had been previously reported in the Pfam database ([1](#_ENREF_1)), but extensive literature searches allowed identification of not only families that were not present in the respective Pfam clan (MBB clan - CL0193), but also families identified as Domains of Unknown function (DUFs). In addition, a total of 15 families are either not present in Pfam or characterized by a Pfam-B model.

Using the formalism of pHMM and adopting the Pfam classification system allows the curators to follow a semi-automated data-retrieval system. First, a family is identified through literature searches; then, the pHMM models are built and compared against Pfam, and finally, the family members are added in the database. This scheme presents more complete classification and accurate annotation concerning β-barrel proteins, due to the added value of the manual annotation and the detailed literature references provided. A comparison of OMPdb against other specific databases that contain β-barrel proteins, reveals that it excels in all aspects; it possesses the most complete and exclusive data for β-barrels, and it offers the most complete interconnection to other public databases, literature references, prediction tools and sequence annotations. OMPdb is working in collaboration with the curators of TCDB ([104](#_ENREF_104)) and Pfam in order to achieve interconnection of the databases by cross-linking the families and keeping the information up to date. This was, at least in part, motivated by the policy meeting.

The database possesses a user-friendly environment through which the user may access the necessary information and cross-references. The underlying level of the database is based on a MySQL system, whereas the upper layer is an Apache-PHP application server. Even though the hierarchy of the database is rather simple, the database is stored in MySQL in order to facilitate advanced queries and easy updates. The web interface of OMPdb offers the user the ability not only to view the available data, but also to submit advanced queries for text searches within the database's protein entries or perform protein or domain searches against OMPdb's proteins or collection of pHMMs, respectively. Raw text, XML and FASTA files of the database can also be downloaded.

Currently, there are only two scientists working on a part-time basis to maintain OMPdb. However, OMPdb is tightly bound to other projects, especially those relevant to the development of prediction methods for β-barrel outer membrane proteins ([105-107](#_ENREF_105)). The existence of a large and reliable data set of β-barrels can be used for large-scale analyses concerning the classification accuracy of existing predictors, for training new prediction methods and for comparative modeling approaches. Our long-term goal is to keep OMPdb as up-to-date as possible, following the regular updates of UniProt and searching the literature for novel experimentally verified β-barrel proteins. Similar to other databases, OMPdb is an ongoing project, and interaction with the user community is vital for its development and refinement.

**PASS2 (Protein Alignments organized as Structural Superfamilies)**

Multiple sequence alignment of highly diverged members of a protein superfamily is non-trivial owing to low sequence identity. Structural properties such as backbone conformational states are employed to drive the alignment using programs like COMPARER ([108](#_ENREF_108)). Previous versions of PASS2 ([109-111](#_ENREF_109)) started off as an HTML-based version, whereas the current version of PASS2 (PASS2.4) ([111](#_ENREF_111)), works on a MYSQL platform with a PHP-front end (<http://caps.ncbs.res.in/pass2/>). This version of the database, that is in direct correspondence with the SCOP 1.75 release ([112](#_ENREF_112)) for the definition of superfamily members, currently offers structure-based sequence alignments of 1961 superfamilies.

One of the key challenges in maintaining and updating the PASS2 database, in response to the accumulation of additional members and superfamilies, is to introduce maximal automation and to reduce manual intervention, without compromising on quality of the data. This is indeed tricky, since evolution brings with it diversification, and this means there could be several 'outliers' ([111](#_ENREF_111)) which are hard to retain during automatic modes of alignments of protein domain superfamilies. Explicit discussions on functional residue variation in a controlled vocabulary within the structure determination reports could enable early recognition of outliers.

**The Structure-Function Linkage Database**

The Structure-Function Linkage Database (SFLD; <http://sfld.rbvi.ucsf.edu/django/>) ([113](#_ENREF_113),[114](#_ENREF_114)) provides a hierarchical classification of functionally diverse enzyme superfamilies that relates the sequence and structural features in each to their constituent enzymes. It was developed to facilitate a conceptual understanding of how the many different reactions represented in such superfamilies evolved ([115](#_ENREF_115)). SFLD is unique among protein annotation resources in its primary focus on this “chemistry-constrained” model ([116](#_ENREF_116)) as a foundation for relating sequence, structural, and catalytic features.

{Placeholder Fig. S3}

Many such superfamilies exist in nature and are estimated to represent at least a third of all enzyme superfamilies ([117](#_ENREF_117)). All members of a given superfamily conserve constellations of functionally important active site residues, while their substrates, products and even overall reactions can be substantially different. At the top level of the hierarchy (superfamily level), SFLD links these conserved active site motifs with chemical features shared by all members. For example, for the enolase superfamily hierarchy (Fig. S3, left panel), all of the characterized member enzymes share a similar active site architecture (Fig. S3, top right; each color represents a different enzyme structure) associated with a specific partial reaction, abstraction of a proton alpha to a substrate carboxylate, and formation of a common type of enolate anion intermediate ([118](#_ENREF_118),[119](#_ENREF_119)). Analysis of sequence patterns across the more than 39,000 divergent member sequences of this superfamily shows that all share variations of this active site architecture, allowing inference about this common catalytic capability. Thus, assignment of a protein of unknown function (“unknown”), to the superfamily constrains the search space for inference of its functional properties to those conserved at the superfamily level. Greater specificity of functional assignment can then be inferred by assignment of sequences to a subgroup or reaction family, as evidence warrants. Reaction families represent sequences experimentally determined or computationally annotated to catalyze a specific overall chemical reaction. They are defined only when sufficient active site information is available from experimental data to distinguish that family from every other in the superfamily (Fig. S3, bottom right; specific residues (cyan) distinguish the dipeptide epimerase family from those common to all other sequences in the superfamily (yellow)).

A major advantage of this classification scheme is its use in addressing problems of "over annotation" and misannotation. For example, annotations found in public databases often assign sequences to a specific reaction when good supporting evidence may be available only for assignment of those proteins to a particular superfamily or subgroup ([120](#_ENREF_120)). Conversely, annotations may be more general than is warranted by the available evidence. Thus, a common annotation in Genbank for peroxiredoxin is "thiol peroxidase" or "redoxin," instead of the functionally specific annotation of Prx5 or Prx6 ([121](#_ENREF_121)).

The database provides high quality manual curation of functional properties at the superfamily, subgroup and family levels for a small set of large and diverse superfamilies (Core SFLD), along with many types of relevant metadata and analysis results. The data and information for each of the superfamily, subgroup, and family levels are all freely available for download via a sophisticated graphical user interface. The available materials include an archive of superfamily sequences, HMMs, annotated multiple sequence alignments, views of 3D structures and annotated active site views that can be opened and manipulated using the freely available Chimera software ([122](#_ENREF_122)), and links to many other relevant resources. An Extended SFLD section provides less curated information for a larger set of functionally diverse enzyme superfamilies.

The SFLD ontology, the Enzyme Structure Function Ontology (ESFO) (in preparation) and a sophisticated MySQL schema codify relationships at multiple levels of granularity, illustrated in Fig. S3. In order for the data to be viewed by users, the SFLD utilizes Django, a high-level Python Web framework, to create the internet-based frontend. Using such a framework makes it significantly easier to develop the interface that users interact with, and also for curators to enter data using a sophisticated graphical user interface that also allows for error checking at point of data entry.

As sequence data continue to increase at a near logarithmic rate, superfamilies have rapidly become larger, in some cases approaching over 100,000 member sequences. To address this challenge and provide support for application of the SFLD hierarchical model to these large and diverse superfamilies of the Core and Extended SFLD, we use protein similarity networks ([123](#_ENREF_123)). These enable interactive exploration of sequence, structure, function relationships defined by our curators and as obtained from other large protein database resources. These networks are generated from all-by-all BLAST ([65](#_ENREF_65)) comparisons of sequence sets at each of the superfamily, subgroup, and family levels and can be visualized using the freely available Cytoscape software ([124](#_ENREF_124)). Many types of attributes that include SFLD curation data and other useful metadata can be mapped to the networks for analysis or visualization. The networks are provided for download as either full sets of sequences (for families and smaller subgroups of the Core SFLD) or sets of representative sequences (representing all the sequences in larger subgroups or superfamilies). Both types of network are currently packaged for download in XGMML format and include a README file, a list of accompanying attributes and files providing basic network statistics. Representative networks are generated using specialized software written to provide summary views of superfamily sequence relationships in a way that preserves access to information about all the sequences making up each representative node ([125](#_ENREF_125)). For the Extended SFLD, only superfamily level networks are provided.

Major challenges confronting the SFLD are similar to those for many other specialist protein databases and include the challenge of obtaining continued funding for development of new and faster analysis tools and to support expansion of coverage of the enzyme superfamily universe. With the rapid growth of the data, new strategies are needed for greater automation of curation without loss of quality.

**TCDB**

The strengths of relational (structured query language, SQL) databases include the multiple means by which data can be structured and queried and their capacity to interface with other systems ([126](#_ENREF_126),[127](#_ENREF_127)). The Transporter Classification Database (TCDB; [www.tcdb.org](http://www.tcdb.org); ([104](#_ENREF_104))) is a relational (Oracle MySQL) database with a PHP front end, currently housed at the San Diego Supercomputer Center ([www.sdsc.edu](http://www.sdsc.edu)). It is a non-redundant database of transport systems from all living organisms, classified according to class, subclass, family, subfamily and system. Transport systems can consist of single proteins or be multi-component, having maximally up to about 100 proteins per system. TCDB currently contains 7 classes, 60 superfamilies, 944 families, 9227 systems, 11806 proteins and 10303 literature references. Systems are entered with exponentially increasing frequency in order of published characterization for over 20 years, and all entries are manually curated. The TC system is based on both function (class and subclass) and phylogeny (family, subfamily and superfamily). It is the only IUBMB-approved system currently in use for transmembrane molecular transport systems ([36-38](#_ENREF_36),[128](#_ENREF_128)).

The TCDB’s management system has multiple benefits:

1. Maintaining a MySQL database enforces a basic controlled structure for each entry, limiting the use of free text and forcing the curator to consider standardized balanced use of features for each entry.
2. TCDB has an administrative system which facilitates maintenance and curation of the MySQL database, including “staff log” pages that can be used to recreate the database at different points in time and prevent vandalism.
3. Use of a MySQL format allows separation between the actual data and the user interface and opened the interoperability (programmatic access) with scripting languages such as Python modules, improving the parsability of the data.
4. The separation of the user interface from database administration introduces the possibility of storing complex or space-consuming relations between data that could have been inconvenient to mirror in a website format (e.g. the superfamily hyperlink).
5. The database can be searched using custom scripts that search the data in a biologically intuitive way.
6. TCDB is associated with numerous software tools to facilitate use, determine topology, establish homology, etc. ([129](#_ENREF_129),[130](#_ENREF_130)).

Although TCDB reminds one of a pre-relational hierarchical database, its implementation as a relational database, subject to a post-relational database management system, allows it to operate as a key-value or document-oriented database. Not Only SQL (NoSQL) and Extensible Markup Language (XML) approaches have several known advantages over relational management systems in terms of query speed and scalability, respectively, especially for clinical data ([131](#_ENREF_131)). In particular, NoSQL is preferred for data where each informational element is discrete and not significantly related to other data. A phonebook or Google Drive might be candidates for NoSQL, storing disparate files.

Using a relational database supports the basic goals of TCDB by organizing knowledge. Though the families are hierarchical, this hierarchy can be represented by “relations”. A hierarchical database makes it more difficult to multiply classify and cross-reference sequences. Thus, the relational system is a good choice for many databases.

The TC system is conceptually similar to an ontology in that it constitutes a “controlled vocabulary” on the sequence level, and the TC system is in concept similar to Wikipedia. It can use crowdsourcing for expansion to optimally inform the research community. New approaches to extend the MySQL architecture, including Pfam integration, development of an ontological system, and Wikification are under consideration (see below).

The use of a relational database management system lends itself to the creation of ontological systems, for example for substrates, inhibitors and activators (e.g., using ChEBI), processes (e.g., using Gene Ontology (GO)), and organismal types (e.g., using UniProt). We believe that there is an important distinction between “classical curation” work that requires a biologist and other management tasks that can be automated to a point but may still require immense manual effort. For example, the setting up of an ontological system for a database such as TCDB requires the development of software to search the database for terms in existing databases such as Chemical Entities of Biological Interest (ChEBI), GO and UniProt that satisfies the needs of the Specialist Protein Resource (SPR). This process will involve the development of a controlled vocabulary and requires cooperation between the SPR and the generalized ontological systems.

Database integration is another highly challenging aspect of database maintenance that can reveal differences in philosophy and definitions between overlapping databases. In some cases, such as in the case of TCDB and Pfam, there are differences in the methods used to establish homology. This fact allows two distinct methods to be used to confirm or refute the conclusions of homology, one using statistical analyses and the other using family specific conserved motifs (HMMs). This is not to imply that Pfam does not use statistical analyses (they use a statistical representation of their alignments), but Pfam does not have absolute or universally applicable homology criteria for membrane proteins as does TCDB; instead Pfam uses family-specific gathering thresholds that are set by the curators using their experience. In this way, both databases can benefit from the approaches and expertise of the other ([132](#_ENREF_132)).

**TIGRFAMs**

The best automated genome annotation pipelines still give unsatisfactory results, inferior to what experienced human curators could achieve gene by gene if given sufficient time. TIGRFAMs (http://www.jcvi.org/cgi-bin/tigrfams/index.cgi) arose to serve the needs of bacterial genome annotation, and to create in each model a tool that reproduces as closely as possible the decisions a manual curator would make ([133](#_ENREF_133)). Building each hidden Markov model involved selecting seed sequences for multiple alignment, trimming away uninformative regions, reviewing the literature, and consulting molecular phylogenetic trees, conserved gene neighborhoods, and whether each candidate functional assignment fits the rest of the inferred metabolic context, as by completing a pathway. The result was a collection of protein family classifiers with only moderate coverage but high reliability. TIGRFAMs’ implementation within the relational database framework simplified its use by genome annotation software that integrated more than a dozen types of computed evidence.

Changes in sources of funding and the coexistence with other sources of evidence for annotation pipelines, especially Pfam ([1](#_ENREF_1)) and FIGfams ([134](#_ENREF_134)), steered TIGRFAMs toward specialization on underserved families of proteins, and eventually, toward discovery work. During 16 years of TIGRFAMs development, the number of available complete microbial genomes grew from less than 20 to more than 2000, moving comparative genomics from its infancy toward maturity as a research technique ([135](#_ENREF_135)). Running each TIGRFAMs HMM against a genome, or a set of several HMMs for the key markers of a subsystem, shows whether or not the molecular markers they defines are present. Each finding that all the markers of a pathway were jointly present, or jointly absent, could be asserted as a Genome Property ([136](#_ENREF_136)). As long as a genome is complete, the absence of evidence for a subsystem equates to good evidence of its absence. Models developed for TIGRFAMs and Genome Properties eventually showed that there are multiple subtypes of CRISPR systems ([137](#_ENREF_137)), that many families of enzymes occur only in bacteria that produce the cofactor F_420_ ([138](#_ENREF_138)), that many short open reading frames actually represent families of small peptides modified post-translationally by radical SAM enzymes ([139](#_ENREF_139)), and that there exist many classes of short, hydrophobic C-terminal regions in bacteria and archaea that act as protein-sorting signals ([140](#_ENREF_140),[141](#_ENREF_141)). For each of these studies, comparative genomics and biocuration work combined to describe new protein families that had no prior description in the literature. The associated publications came after, not before, the families’ additions to TIGRFAMs.

**A protocol to start a database**

*The decision to create a database.*

Many of the SPR databases currently in use were created before the advent of structured query languages or web-content had a low barrier-to-entry. This has changed resulting in the proliferation of databases. Establishing a new database comes with new challenges, for example, deciding whether to start a new database or contribute to an existing one. These options may not be readily apparent - it is still difficult to find specialized databases (the NAR database issue and the associated bioinformatics links directory notwithstanding) and even when found, it is difficult to identify the useful features. Large databases (such as those hosted by NCBI and the EBI) and model organism databases are rich sources of expertise. In some cases, these databases may even share data mining and curation tools. In other cases, databases may include specialized resources as future partners on grant applications; thus addressing one of the recurrent problems of all databases – funding (see Table S2; also discussed in a separate editorial).

*Getting the data structure right.*

Several databases mention the development of more rigorous data structures and controlled vocabularies after the initial launch of the database. This step is arguably one of the most important, even before choosing a storage and distribution technology. In this meeting report, we present our SPR databases are presented and discussed, and our experiences and efforts are described setting the expectation that data structures will evolve over time. Planning for extensibility is perhaps the most important principle, and carefully constructing controlled vocabularies and using of existing ontologies can save work later down the line. Resources and support groups are valuable.

*Storage and distribution technologies.*

Many databases started off as Excel worksheets but subsequently incorporated data modelling and controlled vocabularies. Such tables can be easily converted to tab-delimited files, posted to an FTP site and imported into a MySQL database. In fact, the recent solr4 indexing system is sufficient to convert such files to a NoSQL database that can be accessed by a remote API or via a web-interface written in Javascript. This architecture has its limitations but is especially suited to certain use cases, especially where there are few developer resources or the number of contributing annotation groups is large.

*Interface solutions.*

Relational databases allow the distribution of data and the organization of knowledge according to multiple simultaneous criteria. Sequences can have multiple names and fall into multiple groups with each being stored in hierarchical relationships. Many databases that were historically HTML frame-based websites have been transitioned to relational management systems. Using a relational DB schema enforces a basic controlled structure for each entry, regardless of the entry, similar to filling out a form or questionnaire, in which fields can be left blank. It influences the way data are entered and makes the process more objective, less user-biased. Having a MySQL database also makes it easier to produce releases and backups. Applications range from trivial sequence retrieval to the ability to “stamp” machine annotations directly onto entries. Many tools and links would be difficult to implement without the intelligent MySQL architecture.

*When a SAB should be considered.*

Any long-standing database should have a scientific advisory board (SAB). The SAB could include members of the scientific community with expertise in related areas such as computer science, protein function and structure, and database management. As no manager can possess knowledge of all of these disciplines, communication between these experts is essential for optimal technical and biological modernization. The existence of an SAB also facilitates continuity of the database as members of the SAB can provide important input into grant applications and are likely candidates for future annotation heritage.

**Conclusions**

In conclusion, the SPR meeting in Hinxton brought together expert curators of specialized protein databases. Technical and functional aspects of DB management and development were the focus of this meeting. It emphasized the importance of human curation in the semi-automated system of data presentation, taking advantage of hierarchical systems of biological classification. The benefits of using relational DBs were recognized both with respect to maintenance of controlled and balanced structure and for purposes of interfacing with other DBs. The capacity to expand in new directions commensurate with scientific advances is essential. Equally important will be the implementation of novel technologies as these become developed. We hope that the information provided in this meeting report will facilitate the development of new databases and new DB technologies.

**Conflict of interest statement**

The authors whose names are listed on this editorial certify that they have NO affiliations with or involvement in any organization or entity that constitute a conflict of interest.

**Supplementary Tables and Figures**

***Table S1.*** *Comparative overview of features of specialized protein resources (SPRs), described in this article. These are primarily sequence-centric classification resources.*

Footnotes to Table S1: UniProt ([4](#_ENREF_4)), <http://www.uniprot.org/>; Pfam ([1](#_ENREF_1)), <http://pfam.xfam.org/>, InterPro ([100](#_ENREF_100)), <http://www.ebi.ac.uk/interpro/>; ChEBI ([142](#_ENREF_142)), <http://www.ebi.ac.uk/chebi/>; GO ([143](#_ENREF_143)), <http://geneontology.org/>; BLAST ([144](#_ENREF_144)), <http://blast.ncbi.nlm.nih.gov/Blast.cgi>; HMMer ([145](#_ENREF_145)), <http://hmmer.janelia.org/>; PDB ([39](#_ENREF_39)), <http://www.rcsb.org/pdb/home/home.do>; RefSeq ([2](#_ENREF_2)), [www.ncbi.nlm.nih.gov/refseq/](http://www.ncbi.nlm.nih.gov/refseq/); SwissProt ([3](#_ENREF_3)), <http://web.expasy.org/docs/swiss-prot_guideline.html>; and the EC system ([36-38](#_ENREF_36)), <http://www.chem.qmul.ac.uk/iubmb/enzyme/>; are referenced as indicated throughout the document.

***Table S2.*** *Protein Resources Funding.*

***Fig. S1.*** *Graphical overview showing the conceptual hierarchy of key data needed to define an enzyme. White terms are those that are used in most protein database and are well defined, e.g. the Gene Ontology defines the Molecular Function, Cellular Component and Biological Process via established ontologies. Blue terms are those that are used routinely in many specialist as well as general enzyme databases, such as MACiE, EzCatDB, UniProtKB, etc. Green terms are unique to MACiE.*

***Fig. S2.*** *An example query with results in MACiE – query MACiE for all entries that utilize hydrogen peroxide as a substrate.*

***Fig. S3.*** *SFLD hierarchical classification scheme.*

**References**

1. Finn, R.D., Bateman, A., Clements, J.*, et al.* (2014) Pfam: the protein families database. *Nucleic Acids Res*, **42**, D222-230.

2. Pruitt, K.D., Tatusova, T., Brown, G.R.*, et al.* (2012) NCBI Reference Sequences (RefSeq): current status, new features and genome annotation policy. *Nucleic Acids Res*, **40**, D130-135.

3. Stockinger, H., Altenhoff, A.M., Arnold, K.*, et al.* (2014) Fifteen years SIB Swiss Institute of Bioinformatics: life science databases, tools and support. *Nucleic Acids Res*, **42**, W436-441.

4. UniProt-Consortium (2014) Activities at the Universal Protein Resource (UniProt). *Nucleic Acids Res*, **42**, D191-198.

5. Cantarel, B.L., Coutinho, P.M., Rancurel, C.*, et al.* (2009) The Carbohydrate-Active EnZymes database (CAZy): an expert resource for Glycogenomics. *Nucleic Acids Res*, **37**, D233-238.

6. Lombard, V., Golaconda Ramulu, H., Drula, E.*, et al.* (2014) The carbohydrate-active enzymes database (CAZy) in 2013. *Nucleic Acids Res*, **42**, D490-495.

7. Henrissat, B. (1991) A classification of glycosyl hydrolases based on amino acid sequence similarities. *Biochem J*, **280 ( Pt 2)**, 309-316.

8. Campbell, J.A., Davies, G.J., Bulone, V.*, et al.* (1997) A classification of nucleotide-diphospho-sugar glycosyltransferases based on amino acid sequence similarities. *Biochem J*, **326 ( Pt 3)**, 929-939.

9. Akondi, K.B., Muttenthaler, M., Dutertre, S.*, et al.* (2014) Discovery, synthesis, and structure-activity relationships of conotoxins. *Chem Rev*, **114**, 5815-5847.

10. Terlau, H., Olivera, B.M. (2004) Conus venoms: a rich source of novel ion channel-targeted peptides. *Physiol Rev*, **84**, 41-68.

11. Davis, J., Jones, A., Lewis, R.J. (2009) Remarkable inter- and intra-species complexity of conotoxins revealed by LC/MS. *Peptides*, **30**, 1222-1227.

12. Biggs, J.S., Watkins, M., Puillandre, N.*, et al.* (2010) Evolution of Conus peptide toxins: analysis of Conus californicus Reeve, 1844. *Mol Phylogenet Evol*, **56**, 1-12.

13. Chang, D., Duda, T.F., Jr. (2012) Extensive and continuous duplication facilitates rapid evolution and diversification of gene families. *Mol Biol Evol*, **29**, 2019-2029.

14. Puillandre, N., Koua, D., Favreau, P.*, et al.* (2012) Molecular phylogeny, classification and evolution of conopeptides. *J Mol Evol*, **74**, 297-309.

15. Duda, T.F., Jr., Lee, T. (2009) Ecological release and venom evolution of a predatory marine snail at Easter Island. *PLoS One*, **4**, e5558.

16. Duda, T.F., Jr., Chang, D., Lewis, B.D.*, et al.* (2009) Geographic variation in venom allelic composition and diets of the widespread predatory marine gastropod Conus ebraeus. *PLoS One*, **4**, e6245.

17. Kaas, Q., Yu, R., Jin, A.H.*, et al.* (2012) ConoServer: updated content, knowledge, and discovery tools in the conopeptide database. *Nucleic Acids Res*, **40**, D325-330.

18. Kaas, Q., Westermann, J.C., Craik, D.J. (2010) Conopeptide characterization and classifications: an analysis using ConoServer. *Toxicon*, **55**, 1491-1509.

19. Benson, D.A., Clark, K., Karsch-Mizrachi, I.*, et al.* (2014) GenBank. *Nucleic Acids Res*.

20. Berman, H., Henrick, K., Nakamura, H.*, et al.* (2007) The worldwide Protein Data Bank (wwPDB): ensuring a single, uniform archive of PDB data. *Nucleic Acids Res*, **35**, D301-303.

21. Craik, D.J. (2006) Chemistry. Seamless proteins tie up their loose ends. *Science*, **311**, 1563-1564.

22. Kedarisetti, P., Mizianty, M.J., Kaas, Q.*, et al.* (2014) Prediction and characterization of cyclic proteins from sequences in three domains of life. *Biochim Biophys Acta*, **1844**, 181-190.

23. Trabi, M., Craik, D.J. (2002) Circular proteins--no end in sight. *Trends Biochem Sci*, **27**, 132-138.

24. Poth, A.G., Chan, L.Y., Craik, D.J. (2013) Cyclotides as grafting frameworks for protein engineering and drug design applications. *Biopolymers*, **100**, 480-491.

25. Wang, C.K., Kaas, Q., Chiche, L.*, et al.* (2008) CyBase: a database of cyclic protein sequences and structures, with applications in protein discovery and engineering. *Nucleic Acids Res*, **36**, D206-210.

26. Lenfant, N., Hotelier, T., Bourne, Y.*, et al.* (2013) Proteins with an alpha/beta hydrolase fold: Relationships between subfamilies in an ever-growing superfamily. *Chem Biol Interact*, **203**, 266-268.

27. Lenfant, N., Hotelier, T., Velluet, E.*, et al.* (2013) ESTHER, the database of the alpha/beta-hydrolase fold superfamily of proteins: tools to explore diversity of functions. *Nucleic Acids Res*, **41**, D423-429.

28. Marchot, P., Chatonnet, A. (2012) Enzymatic activity and protein interactions in alpha/beta hydrolase fold proteins: moonlighting versus promiscuity. *Protein Pept Lett*, **19**, 132-143.

29. Lenfant, N., Hotelier, T., Bourne, Y.*, et al.* (2014) Tracking the origin and divergence of cholinesterases and neuroligins: the evolution of synaptic proteins. *J Mol Neurosci*, **53**, 362-369.

30. Cousin, X., Hotelier, T., Lievin, P.*, et al.* (1996) A cholinesterase genes server (ESTHER): a database of cholinesterase-related sequences for multiple alignments, phylogenetic relationships, mutations and structural data retrieval. *Nucleic Acids Res*, **24**, 132-136.

31. Renault, L., Negre, V., Hotelier, T.*, et al.* (2005) New friendly tools for users of ESTHER, the database of the alpha/beta-hydrolase fold superfamily of proteins. *Chem Biol Interact*, **157-158**, 339-343.

32. Hotelier, T., Negre, V. (2010) Insecticide resistance through mutations in cholinesterases or carboxylesterases: data mining in the ESTHER database. *J. Pestic Sci.*, **35**, 315-320.

33. Chatonnet, A., Cousin, X., Robinson, A. (2001) Links between kinetic data and sequences in the alpha/beta-hydrolases fold database. *Brief Bioinform*, **2**, 30-37.

34. Nagano, N. (2005) EzCatDB: the Enzyme Catalytic-mechanism Database. *Nucleic Acids Res*, **33**, D407-412.

35. Nagano, N., Nakayama, N., Ikeda, K.*, et al.* (2015) EzCatDB: the enzyme reaction database, 2015 update. *Nucleic Acids Res*, **43**, D453-458.

36. Tipton, K.F. (1994) Nomenclature Committee of the International Union of Biochemistry and Molecular Biology (NC-IUBMB). Enzyme nomenclature. Recommendations 1992. Supplement: corrections and additions. *Eur J Biochem*, **223**, 1-5.

37. Fleischmann, A., Darsow, M., Degtyarenko, K.*, et al.* (2004) IntEnz, the integrated relational enzyme database. *Nucleic Acids Res*, **32**, D434-437.

38. McDonald, A.G., Boyce, S., Tipton, K.F. (2009) ExplorEnz: the primary source of the IUBMB enzyme list. *Nucleic Acids Res*, **37**, D593-597.

39. Rose, P.W., Bi, C., Bluhm, W.F.*, et al.* (2013) The RCSB Protein Data Bank: new resources for research and education. *Nucleic Acids Res*, **41**, D475-482.

40. Cuff, A.L., Sillitoe, I., Lewis, T.*, et al.* (2011) Extending CATH: increasing coverage of the protein structure universe and linking structure with function. *Nucleic Acids Res*, **39**, D420-426.

41. Kanehisa, M., Goto, S., Sato, Y.*, et al.* (2014) Data, information, knowledge and principle: back to metabolism in KEGG. *Nucleic Acids Res*, **42**, D199-205.

42. McEntyre, J., Lipman, D. (2001) PubMed: bridging the information gap. *CMAJ*, **164**, 1317-1319.

43. Bockaert, J., Pin, J.P. (1999) Molecular tinkering of G protein-coupled receptors: an evolutionary success. *EMBO J.*, **18**, 1723-1729.

44. Lagerstrom, M.C., Schioth, H.B. (2008) Structural diversity of G protein-coupled receptors and significance for drug discovery. *Nature reviews. Drug discovery*, **7**, 339-357.

45. Overington, J.P., Al-Lazikani, B., Hopkins, A.L. (2006) How many drug targets are there? *Nature reviews. Drug discovery*, **5**, 993-996.

46. Garland, S.L. (2013) Are GPCRs Still a Source of New Targets? *Journal of biomolecular screening*, **18**, 947-966.

47. Isberg, V., Vroling, B., van der Kant, R.*, et al.* (2014) GPCRDB: an information system for G protein-coupled receptors. *Nucleic Acids Res*, **42**, D422-425.

48. Katritch, V., Cherezov, V., Stevens, R.C. (2013) Structure-function of the G protein-coupled receptor superfamily. *Annu. Rev. Pharmacol. Toxicol.*, **53**, 531-556.

49. Isberg, V., de Graaf, C., Bortolato, A.*, et al.* (2015) Generic GPCR residue numbers - aligning topology maps while minding the gaps. *Trends Pharmacol Sci*, **36**, 22-31.

50. Pawson, A.J., Sharman, J.L., Benson, H.E.*, et al.* (2014) The IUPHAR/BPS Guide to PHARMACOLOGY: an expert-driven knowledgebase of drug targets and their ligands. *Nucleic Acids Res*, **42**, D1098-1106.

51. Harmar, A.J., Hills, R.A., Rosser, E.M.*, et al.* (2009) IUPHAR-DB: the IUPHAR database of G protein-coupled receptors and ion channels. *Nucleic Acids Res*, **37**, D680-685.

52. Alexander, S.P., Mathie, A., Peters, J.A. (2011) Guide to Receptors and Channels (GRAC), 5th edition. *Br J Pharmacol*, **164 Suppl 1**, S1-324.

53. Gaulton, A., Bellis, L.J., Bento, A.P.*, et al.* (2012) ChEMBL: a large-scale bioactivity database for drug discovery. *Nucleic Acids Res*, **40**, D1100-1107.

54. (2014) UniProt: a hub for protein information. *Nucleic Acids Res*.

55. Cunningham, F., Amode, M.R., Barrell, D.*, et al.* (2015) Ensembl 2015. *Nucleic Acids Res*, **43**, D662-669.

56. Bolton E, Wang Y, Thiessen PA*, et al.* (2008) Integrated Platform of Small Molecules and Biological Activities. *Annual Reports in Computational Chemistry*, **4**, 217-240.

57. Law, V., Knox, C., Djoumbou, Y.*, et al.* (2014) DrugBank 4.0: shedding new light on drug metabolism. *Nucleic Acids Res*, **42**, D1091-1097.

58. Alexander, S.P., Benson, H.E., Faccenda, E.*, et al.* (2013) The Concise Guide to PHARMACOLOGY 2013/14: overview. *Br J Pharmacol*, **170**, 1449-1458.

59. Spedding, M. (2011) Resolution of controversies in drug/receptor interactions by protein structure. Limitations and pharmacological solutions. *Neuropharmacology*, **60**, 3-6.

60. Tough, D.F., Lewis, H.D., Rioja, I.*, et al.* (2014) Epigenetic pathway targets for the treatment of disease: accelerating progress in the development of pharmacological tools: IUPHAR Review 11. *Br J Pharmacol*, **171**, 4981-5010.

61. Bonner, T.I. (2014) Should pharmacologists care about alternative splicing? IUPHAR Review 4. *Br J Pharmacol*, **171**, 1231-1240.

62. Christopoulos, A., Changeux, J.P., Catterall, W.A.*, et al.* (2014) International union of basic and clinical pharmacology. XC. multisite pharmacology: recommendations for the nomenclature of receptor allosterism and allosteric ligands. *Pharmacol Rev*, **66**, 918-947.

63. Baxevanis, A.D., Landsman, D. (1996) Histone Sequence Database: a compilation of highly-conserved nucleoprotein sequences. *Nucleic Acids Res*, **24**, 245-247.

64. Baxevanis, A.D., Arents, G., Moudrianakis, E.N.*, et al.* (1995) A variety of DNA-binding and multimeric proteins contain the histone fold motif. *Nucleic Acids Res*, **23**, 2685-2691.

65. Altschul, S.F., Madden, T.L., Schaffer, A.A.*, et al.* (1997) Gapped BLAST and PSI-BLAST: a new generation of protein database search programs. *Nucleic Acids Res*, **25**, 3389-3402.

66. Eddy, S.R. (2009) A new generation of homology search tools based on probabilistic inference. *Genome Inform*, **23**, 205-211.

67. Marino-Ramirez, L., Levine, K.M., Morales, M.*, et al.* (2011) The Histone Database: an integrated resource for histones and histone fold-containing proteins. *Database (Oxford)*, **2011**, bar048.

68. Sondermann, H., Soisson, S.M., Bar-Sagi, D.*, et al.* (2003) Tandem histone folds in the structure of the N-terminal segment of the ras activator Son of Sevenless. *Structure*, **11**, 1583-1593.

69. Rose, P.W., Prlic, A., Bi, C.*, et al.* (2014) The RCSB Protein Data Bank: views of structural biology for basic and applied research and education. *Nucleic Acids Res*.

70. Talbert, P.B., Ahmad, K., Almouzni, G.*, et al.* (2012) A unified phylogeny-based nomenclature for histone variants. *Epigenetics Chromatin*, **5**, 7.

71. Manning, G., Whyte, D.B., Martinez, R.*, et al.* (2002) The protein kinase complement of the human genome. *Science*, Vol. 298, pp. 1912-1934.

72. Goldberg, J.M., Manning, G., Liu, A.*, et al.* (2006) The dictyostelium kinome--analysis of the protein kinases from a simple model organism. *PLoS Genet*, Vol. 2, pp. e38.

73. Caenepeel, S., Charydczak, G., Sudarsanam, S.*, et al.* (2004) The mouse kinome: discovery and comparative genomics of all mouse protein kinases. *Proceedings of the National Academy of Sciences of the United States of America*, **101**, 11707-11712.

74. Bradham, C.A., Foltz, K.R., Beane, W.S.*, et al.* (2006) The sea urchin kinome: a first look. *Developmental biology*, **300**, 180-193.

75. Eisen, J.A., Coyne, R.S., Wu, M.*, et al.* (2006) Macronuclear genome sequence of the ciliate Tetrahymena thermophila, a model eukaryote. *PLoS biology*, **4**, e286.

76. Srivastava, M., Simakov, O., Chapman, J.*, et al.* (2010) The Amphimedon queenslandica genome and the evolution of animal complexity. *Nature*, **466**, 720-726.

77. Stajich, J.E., Wilke, S.K., Ahren, D.*, et al.* (2010) Insights into evolution of multicellular fungi from the assembled chromosomes of the mushroom Coprinopsis cinerea (Coprinus cinereus). *Proceedings of the National Academy of Sciences of the United States of America*, **107**, 11889-11894.

78. Plowman, G.D., Sudarsanam, S., Bingham, J.*, et al.* (1999) The protein kinases of Caenorhabditis elegans: a model for signal transduction in multicellular organisms. *Proceedings of the National Academy of Sciences of the United States of America*, **96**, 13603-13610.

79. Bingham, J., Plowman, G.D., Sudarsanam, S. (2000) Informatics issues in large-scale sequence analysis: elucidating the protein kinases of C. elegans. *Journal of cellular biochemistry*, **80**, 181-186.

80. Manning, G. (2005) Genomic overview of protein kinases. *WormBook : the online review of C. elegans biology*, 1-19.

81. Hunter, T., Plowman, G.D. (1997) The protein kinases of budding yeast: six score and more. *Trends in biochemical sciences*, **22**, 18-22.

82. Manning, G., Plowman, G.D., Hunter, T.*, et al.* (2002) Evolution of protein kinase signaling from yeast to man. *Trends in biochemical sciences*, Vol. 27, pp. 514-520.

83. Krupa, A., Abhinandan, K.R., Srinivasan, N. (2004) KinG: a database of protein kinases in genomes. *Nucleic Acids Res*, **32**, D153-155.

84. Hanks, S.K., Hunter, T. (1995) Protein kinases 6. The eukaryotic protein kinase superfamily: kinase (catalytic) domain structure and classification. *Faseb J*, **9**, 576-596.

85. Gowri, V.S., Krishnadev, O., Swamy, C.S.*, et al.* (2006) MulPSSM: a database of multiple position-specific scoring matrices of protein domain families. *Nucleic Acids Res*, **34**, D243-246.

86. Deshmukh, K., Anamika, K., Srinivasan, N. (2010) Evolution of domain combinations in protein kinases and its implications for functional diversity. *Prog Biophys Mol Biol*, **102**, 1-15.

87. Rakshambikai, R., Gnanavel, M., Srinivasan, N. (2014) Hybrid and rogue kinases encoded in the genomes of model eukaryotes. *PLoS One*, **9**, e107956.

88. Martin, J., Anamika, K., Srinivasan, N. (2010) Classification of protein kinases on the basis of both kinase and non-kinase regions. *PLoS One*, **5**, e12460.

89. Bhaskara, R.M., Mehrotra, P., Rakshambikai, R.*, et al.* (2014) The relationship between classification of multi-domain proteins using an alignment-free approach and their functions: a case study with immunoglobulins. *Mol Biosyst*, **10**, 1082-1093.

90. Gnanavel, M., Mehrotra, P., Rakshambikai, R.*, et al.* (2014) CLAP: a web-server for automatic classification of proteins with special reference to multi-domain proteins. *BMC Bioinformatics*, **15**, 343.

91. Holliday, G.L., Andreini, C., Fischer, J.D.*, et al.* (2012) MACiE: exploring the diversity of biochemical reactions. *Nucleic Acids Res*, **40**, D783-789.

92. Holliday, G.L., Almonacid, D.E., Bartlett, G.J.*, et al.* (2007) MACiE (Mechanism, Annotation and Classification in Enzymes): novel tools for searching catalytic mechanisms. *Nucleic Acids Res*, **35**, D515-520.

93. Rawlings, N.D., Waller, M., Barrett, A.J.*, et al.* (2014) MEROPS: the database of proteolytic enzymes, their substrates and inhibitors. *Nucleic Acids Res*, **42**, D503-509.

94. Barrett, A.J., Rawlings, N.D. (2007) 'Species' of peptidases. *Biol Chem*, **388**, 1151-1157.

95. Boyd, S.E., Pike, R.N., Rudy, G.B.*, et al.* (2005) PoPS: a computational tool for modeling and predicting protease specificity. *J Bioinform Comput Biol*, **3**, 551-585.

96. Song, J., Tan, H., Perry, A.J.*, et al.* (2012) PROSPER: an integrated feature-based tool for predicting protease substrate cleavage sites. *PLoS One*, **7**, e50300.

97. Rawlings, N.D. (2009) A large and accurate collection of peptidase cleavages in the MEROPS database. *Database (Oxford)*, **2009**, bap015.

98. Rawlings, N.D., Morton, F.R., Kok, C.Y.*, et al.* (2008) MEROPS: the peptidase database. *Nucleic Acids Res*, **36**, D320-325.

99. Rawlings, N.D., Barrett, A.J., Bateman, A. (2012) MEROPS: the database of proteolytic enzymes, their substrates and inhibitors. *Nucleic Acids Res*, **40**, D343-350.

100. Mitchell, A., Chang, H.Y., Daugherty, L.*, et al.* (2015) The InterPro protein families database: the classification resource after 15 years. *Nucleic Acids Res*, **43**, D213-221.

101. Rawlings, N.D., Morton, F.R. (2008) The MEROPS batch BLAST: a tool to detect peptidases and their non-peptidase homologues in a genome. *Biochimie*, **90**, 243-259.

102. Lane, L., Argoud-Puy, G., Britan, A.*, et al.* (2012) neXtProt: a knowledge platform for human proteins. *Nucleic Acids Res*, **40**, D76-83.

103. Tsirigos, K.D., Bagos, P.G., Hamodrakas, S.J. (2011) OMPdb: a database of {beta}-barrel outer membrane proteins from Gram-negative bacteria. *Nucleic Acids Res*, **39**, D324-331.

104. Saier, M.H., Jr., Reddy, V.S., Tamang, D.G.*, et al.* (2014) The transporter classification database. *Nucleic Acids Res*, **42**, D251-258.

105. Bagos, P.G., Liakopoulos, T.D., Hamodrakas, S.J. (2005) Evaluation of methods for predicting the topology of beta-barrel outer membrane proteins and a consensus prediction method. *BMC Bioinformatics*, **6**, 7.

106. Bagos, P.G., Liakopoulos, T.D., Spyropoulos, I.C.*, et al.* (2004) PRED-TMBB: a web server for predicting the topology of beta-barrel outer membrane proteins. *Nucleic Acids Res*, **32**, W400-404.

107. Bagos, P.G., Liakopoulos, T.D., Spyropoulos, I.C.*, et al.* (2004) A Hidden Markov Model method, capable of predicting and discriminating beta-barrel outer membrane proteins. *BMC Bioinformatics*, **5**, 29.

108. Sali, A., Blundell, T.L. (1990) Definition of general topological equivalence in protein structures. A procedure involving comparison of properties and relationships through simulated annealing and dynamic programming. *J Mol Biol*, **212**, 403-428.

109. Mallika, V., Bhaduri, A., Sowdhamini, R. (2002) PASS2: a semi-automated database of protein alignments organised as structural superfamilies. *Nucleic Acids Res*, **30**, 284-288.

110. Bhaduri, A., Pugalenthi, G., Sowdhamini, R. (2004) PASS2: an automated database of protein alignments organised as structural superfamilies. *BMC Bioinformatics*, **5**, 35.

111. Gandhimathi, A., Nair, A.G., Sowdhamini, R. (2012) PASS2 version 4: an update to the database of structure-based sequence alignments of structural domain superfamilies. *Nucleic Acids Res*, **40**, D531-534.

112. Murzin, A.G., Brenner, S.E., Hubbard, T.*, et al.* (1995) SCOP: a structural classification of proteins database for the investigation of sequences and structures. *J Mol Biol*, **247**, 536-540.

113. Pegg, S.C., Brown, S.D., Ojha, S.*, et al.* (2006) Leveraging enzyme structure-function relationships for functional inference and experimental design: the structure-function linkage database. *Biochemistry*, **45**, 2545-2555.

114. Akiva, E., Brown, S., Almonacid, D.E.*, et al.* (2014) The Structure-Function Linkage Database. *Nucleic Acids Res*, **42**, D521-530.

115. Gerlt, J.A., Babbitt, P.C. (2001) Divergent evolution of enzymatic function: mechanistically diverse superfamilies and functionally distinct suprafamilies. *Annu Rev Biochem*, **70**, 209-246.

116. Babbitt, P.C., Gerlt, J.A. (1997) Understanding enzyme superfamilies. Chemistry As the fundamental determinant in the evolution of new catalytic activities. *J Biol Chem*, **272**, 30591-30594.

117. Almonacid, D.E., Babbitt, P.C. (2011) Toward mechanistic classification of enzyme functions. *Curr Opin Chem Biol*, **15**, 435-442.

118. Babbitt, P.C., Hasson, M.S., Wedekind, J.E.*, et al.* (1996) The enolase superfamily: a general strategy for enzyme-catalyzed abstraction of the alpha-protons of carboxylic acids. *Biochemistry*, **35**, 16489-16501.

119. Gerlt, J.A., Babbitt, P.C., Rayment, I. (2005) Divergent evolution in the enolase superfamily: the interplay of mechanism and specificity. *Arch Biochem Biophys*, **433**, 59-70.

120. Schnoes, A.M., Brown, S.D., Dodevski, I.*, et al.* (2009) Annotation error in public databases: misannotation of molecular function in enzyme superfamilies. *PLoS Comput Biol*, **5**, e1000605.

121. Nelson, K.J., Knutson, S.T., Soito, L.*, et al.* (2011) Analysis of the peroxiredoxin family: using active-site structure and sequence information for global classification and residue analysis. *Proteins*, **79**, 947-964.

122. Pettersen, E.F., Goddard, T.D., Huang, C.C.*, et al.* (2004) UCSF Chimera--a visualization system for exploratory research and analysis. *J Comput Chem*, **25**, 1605-1612.

123. Atkinson, H.J., Morris, J.H., Ferrin, T.E.*, et al.* (2009) Using sequence similarity networks for visualization of relationships across diverse protein superfamilies. *PLoS One*, **4**, e4345.

124. Shannon, P., Markiel, A., Ozier, O.*, et al.* (2003) Cytoscape: a software environment for integrated models of biomolecular interaction networks. *Genome Res*, **13**, 2498-2504.

125. Barber, A.E., 2nd, Babbitt, P.C. (2012) Pythoscape: a framework for generation of large protein similarity networks. *Bioinformatics*, **28**, 2845-2846.

126. Stein, L. (2013) Creating databases for biological information: an introduction. *Curr Protoc Bioinformatics*, **Chapter 9**, Unit9 1.

127. Jamison, D.C. (2003) Structured Query Language (SQL) fundamentals. *Curr Protoc Bioinformatics*, **Chapter 9**, Unit9 2.

128. Saier, M.H., Jr. (2000) A functional-phylogenetic classification system for transmembrane solute transporters. *Microbiol Mol Biol Rev*, **64**, 354-411.

129. Wakabayashi ST, Shlykov MA, Kumar U*, et al.* (2013) Deducing transport protein evolution based on sequence, structure, and function. *Protein Families: Relating Protein Sequence, Structure, and Function. In: Christine AO, Alex B, editors. 1st edn. Hoboken, NJ: Wiley; 2013.*

130. Reddy, V.S., Saier, M.H., Jr. (2012) BioV Suite--a collection of programs for the study of transport protein evolution. *Febs J*, **279**, 2036-2046.

131. Lee, K.K., Tang, W.C., Choi, K.S. (2013) Alternatives to relational database: comparison of NoSQL and XML approaches for clinical data storage. *Comput Methods Programs Biomed*, **110**, 99-109.

132. Chiang, Z., Vastermark, A., Punta, M.*, et al.* (2015) The complexity, challenges and benefits of comparing two transporter classification systems in TCDB and Pfam. *Brief Bioinform*.

133. Haft, D.H., Selengut, J.D., Richter, R.A.*, et al.* (2013) TIGRFAMs and Genome Properties in 2013. *Nucleic Acids Res*, **41**, D387-395.

134. Meyer, F., Overbeek, R., Rodriguez, A. (2009) FIGfams: yet another set of protein families. *Nucleic Acids Res*, **37**, 6643-6654.

135. Haft, D.H. (2015) Using comparative genomics to drive new discoveries in microbiology. *Curr Opin Microbiol*, **23C**, 189-196.

136. Haft, D.H., Selengut, J.D., Brinkac, L.M.*, et al.* (2005) Genome Properties: a system for the investigation of prokaryotic genetic content for microbiology, genome annotation and comparative genomics. *Bioinformatics*, **21**, 293-306.

137. Haft, D.H., Selengut, J., Mongodin, E.F.*, et al.* (2005) A guild of 45 CRISPR-associated (Cas) protein families and multiple CRISPR/Cas subtypes exist in prokaryotic genomes. *PLoS Comput Biol*, **1**, e60.

138. Selengut, J.D., Haft, D.H. (2010) Unexpected abundance of coenzyme F(420)-dependent enzymes in Mycobacterium tuberculosis and other actinobacteria. *J Bacteriol*, **192**, 5788-5798.

139. Haft, D.H., Basu, M.K. (2011) Biological systems discovery in silico: radical S-adenosylmethionine protein families and their target peptides for posttranslational modification. *J Bacteriol*, **193**, 2745-2755.

140. Haft, D.H., Payne, S.H., Selengut, J.D. (2012) Archaeosortases and exosortases are widely distributed systems linking membrane transit with posttranslational modification. *J Bacteriol*, **194**, 36-48.

141. Haft, D.H., Varghese, N. (2011) GlyGly-CTERM and rhombosortase: a C-terminal protein processing signal in a many-to-one pairing with a rhomboid family intramembrane serine protease. *PLoS One*, **6**, e28886.

142. Hastings, J., de Matos, P., Dekker, A.*, et al.* (2013) The ChEBI reference database and ontology for biologically relevant chemistry: enhancements for 2013. *Nucleic Acids Res*, **41**, D456-463.

143. (2015) Gene Ontology Consortium: going forward. *Nucleic Acids Res*, **43**, D1049-1056.

144. Altschul, S.F., Gish, W., Miller, W.*, et al.* (1990) Basic local alignment search tool. *J Mol Biol*, **215**, 403-410.

145. Finn, R.D., Clements, J., Eddy, S.R. (2011) HMMER web server: interactive sequence similarity searching. *Nucleic Acids Res*, **39**, W29-37.
