# Supplementary material for: Creating a specialist protein resource network: a meeting report for the protein bioinformatics and community resources retreat
Source: Database (Oxford). 2015 Jul 11;2015:bav063. doi: 10.1093/database/bav063 (PMC4499208; doi:10.1093/database/bav063)
Supplement: Supplementary Data [file supp_bav063_suppl_data.zip › Table S1 May14.docx]

*Table S1.* **Comparative overview of features of specialized protein resources (SPRs), described in this article. These are primarily sequence-centric classification resources.**

| **Database name** | **Abbreviation** | **Primary purpose or function** | **Date of inception** | **(Relational) DMS and other technologies** | **Author(s)** |
| --- | --- | --- | --- | --- | --- |
| Carbohydrate-Active enZYmes Database | CAZy | Describes the families of structurally-related catalytic and carbohydrate-binding modules (or functional domains) of enzymes that break down, modify, or build glycosidic bonds | Early 1990s | Transitioned from tab-delimited web-site to MySQL in 1999 | Bernard Henrissat |
| ConoServer | ConoServer | Marine cone snail toxin database | 2008 | MySQL | David Craik and Quentin Kaas |
| CyBASE | CyBASE | Ribosomally synthesized circular proteins | 2008 | MySQL | David Craik and Quentin Kaas |
| ESTerases and alpha/beta-Hydrolase Enzymes and Relatives database | ESTHER | Analysis of proteins belonging to the superfamily of alpha/beta-hydrolases | 1996 | ACeDB system (not MySQL) | Arnaud Chatonnet and Nicolas Lenfant |
| Enzyme reaction database | EzCatDB | Classification of enzyme reactions, along with annotation of active-sites and ligands in PDB data for enzymes | 2004 | PostgreSQL | Nozomi Nagano |
| G protein-coupled receptor database | GPCRDB | G protein-coupled receptor database | 1993 | MySQL | David Gloriam and Vignir Isberg |
| IUPHAR/BPS Guide to PHARMACOLOGY | GtoPdb | Expert-driven curation of pharmacological targets and the substances that act on them | 2011 (builds upon IUPHAR-DB established 2003) | PostgreSQL and Oracle; front end uses Java Servlets, JSP, HTML, CSS, JavaScript and JQuery. | Michael Spedding and Joanna L. Sharman |
| Histone Database | Histone DB | A collection of all histone core proteins, linker histones, and histone fold protein sequences. | 1996 | Oracle with Perl’s Database Interface (DBI) and the Oracle database driver for the DBI module (DBD::Oracle) | David Landsman |
| Protein kinase database | Kinase.com | Function and evolution of protein kinases | 1999 | MySQL with web framework Django | Gerard Manning and Mark Jinan Chen |
| Database of Kinases in Genomes | KinG | KinG is a resource on Ser/Thr/Tyr kinases encoded in the completely sequenced genomes of prokaryotes, viruses and eukaryotes | 2004 | Was developed using Netbeans IDE using Java, JSP, Servlets, AJAX, Jquery, XML, HTML and CSS | Narayanaswamy Srinivasan |
| Mechanism, Annotation and Classification in Enzymes | MACiE | Enzymes, their chemical transformations, reaction mechanisms and catalytic residues | 2002 | MySQL | Gemma L. Holliday |
| The peptidase database | MEROPS | Provides a classification and nomenclature for proteolytic enzymes and the protein and small molecule inhibitors that affect their enzymatic activity | 1996 | MySQL | Neil D. Rawlings |
| neXtProt | neXtProt | Knowledge resource on human proteins | 2011 | PostgreSQL | Amos Bairoch |
| Outer Membrane Protein Database | OMPdb | Classification of bacterial outer membrane proteins | 2005 | MySQL with Apache-PHP upper layer; uses HMMs and offers XML-formatted download | Pantelis G. Bagos and Konstantinos D. Tsirigos |
| Protein alignments organized as structural superfamilies database | PASS2 | Protein sequence Alignments of Structural Superfamilies | 1998 | Began as HTML-based version, and currently works on MySQL platform with PHP-front end | Ramanathan Sowdhamini |
| Structure-Function Linkage Database | SFLD | Hierarchical classification of functionally diverse enzyme superfamilies | 2001 | MySQL with web framework Django | Patricia C. Babbitt and Gemma L. Holliday |
| Transporter Classification Database | TCDB | Classification of transport proteins | 1998 (in MySQL form) | Oracel MySQL 5.7 with a PHP front end, housed by SDSC; InnoDB MySQL table engine (used to be MylSAM), Python | Milton Saier and Ake Vastermark |
| TIGRFAMs database of protein families | TIGRFAMs | Genome annotation pipeline support, comparative genomics, discovery of new subsystems | 2001 | Sybase | Daniel Haft and Jeremy Selengut |
