# Supplementary material for: Creating a specialist protein resource network: a meeting report for the protein bioinformatics and community resources retreat
Source: Database (Oxford). 2015 Jul 11;2015:bav063. doi: 10.1093/database/bav063 (PMC4499208; doi:10.1093/database/bav063)
Supplement: Supplementary Data [file supp_bav063_suppl_data.zip › Table S2 May14.docx]

Table S2. Protein Resources Funding.

| Resource name | FTE staff | Grant funded | Institutional funding | Other funding | Main funding source | Comments |
| --- | --- | --- | --- | --- | --- | --- |
| CAZy | 5.5 | 0 | 0 | 0 |  | 3.5 FTE have permanent positions (no need to search for their salaries); two postdocs paid by research grants |
| ConoServer | 0.1 | 0 | 0 | 0 | No explicit funding |  |
| CyBase | 0.1 | 0 | 0 | 0 | No explicit funding |  |
| ESTHER | 2 | 1 |  |  | ANR |  |
| EzCatDB | 1 | 0 | 0 | 1.5 | Support from METI | 1 FTE + 1 annotator + 0.5 SE (outsourcing IT company for system developments; occasionally, helped by a few more SEs) |
| GPCRDB | 1 | 0 | 1 |  | CMBI |  |
| Guidetopharmacology | 4.5 | 1 | Supported | 2 | Wellcome Trust | Wellcome critical, may have support from professional sociéties |
| Histone database |  |  | Supported |  | NIH (Intramural Research Program of NLM) |  |
| KinBase | 0.2 | 0 | 0.2 | 0 | Genentech | Part time postdoc + after-hours PI |
| KinG | 1 | 0 | 0 | 0 | No explicit funding currently |  |
| MACiE | 0.1 | 0 | 0 | 0 | Shared NSF grant |  |
| MEROPS | 1 | 0 | 1 | 0 | WTSI |  |
| neXtProt | 8.3 | 2.3 | 6 |  | SIB | Lost 3 FTE at end of 2013 because of non-replacement of an industrial contract (number of FTE reported is post-loss ie 2014) |
| OMPdb | 0.2 | 0 | 0 | 0 | No explicit funding |  |
| PASS2 | 2 | 2 | 1 | 1 | Department of Biotechnology- India; NCBS (TIFR) | DBT grant is for five years, maybe extendable for a further three years, but future funding is not clear. |
| SFLD | 4.5 | 2 | 0 | 0 | NIH, NSF | Rising costs of senior people like programmers |
| TCDB | 2 | 3 | 0 | 3 | NIH |  |
| TIGRFAMs* | 0 | 0 | 0 | 0 | NIH, DOE | No longer being developed at JCVI. Leaders have relocated. Future uncertain. |

*= presently a “zombie” (unsupported) database
