# Supplementary figures and images for: Creating a specialist protein resource network: a meeting report for the protein bioinformatics and community resources retreat
Source: Database (Oxford). 2015 Jul 11;2015:bav063. doi: 10.1093/database/bav063 (PMC4499208; doi:10.1093/database/bav063)

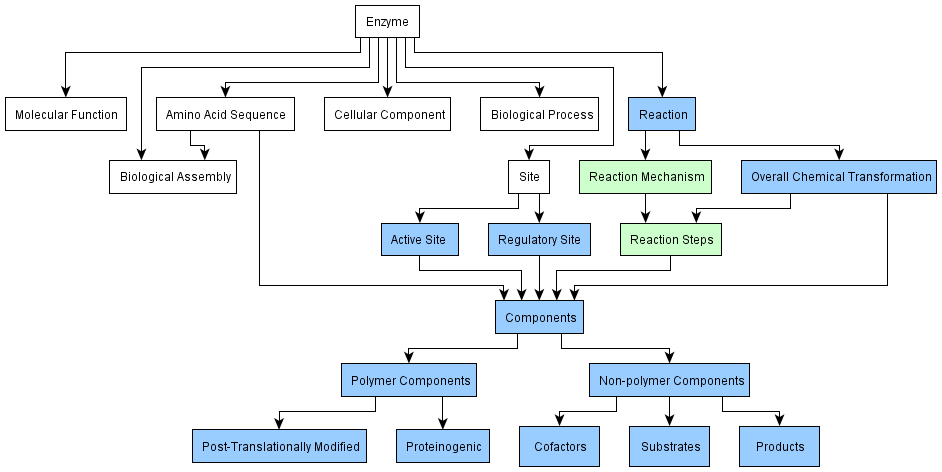

Supplement: Supplementary Data [file supp_bav063_suppl_data.zip › Fig S1 May14.png]

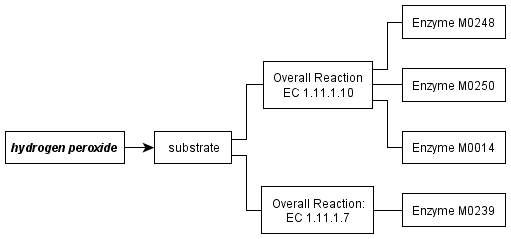

Supplement: Supplementary Data [file supp_bav063_suppl_data.zip › Fig S2 May14.png]

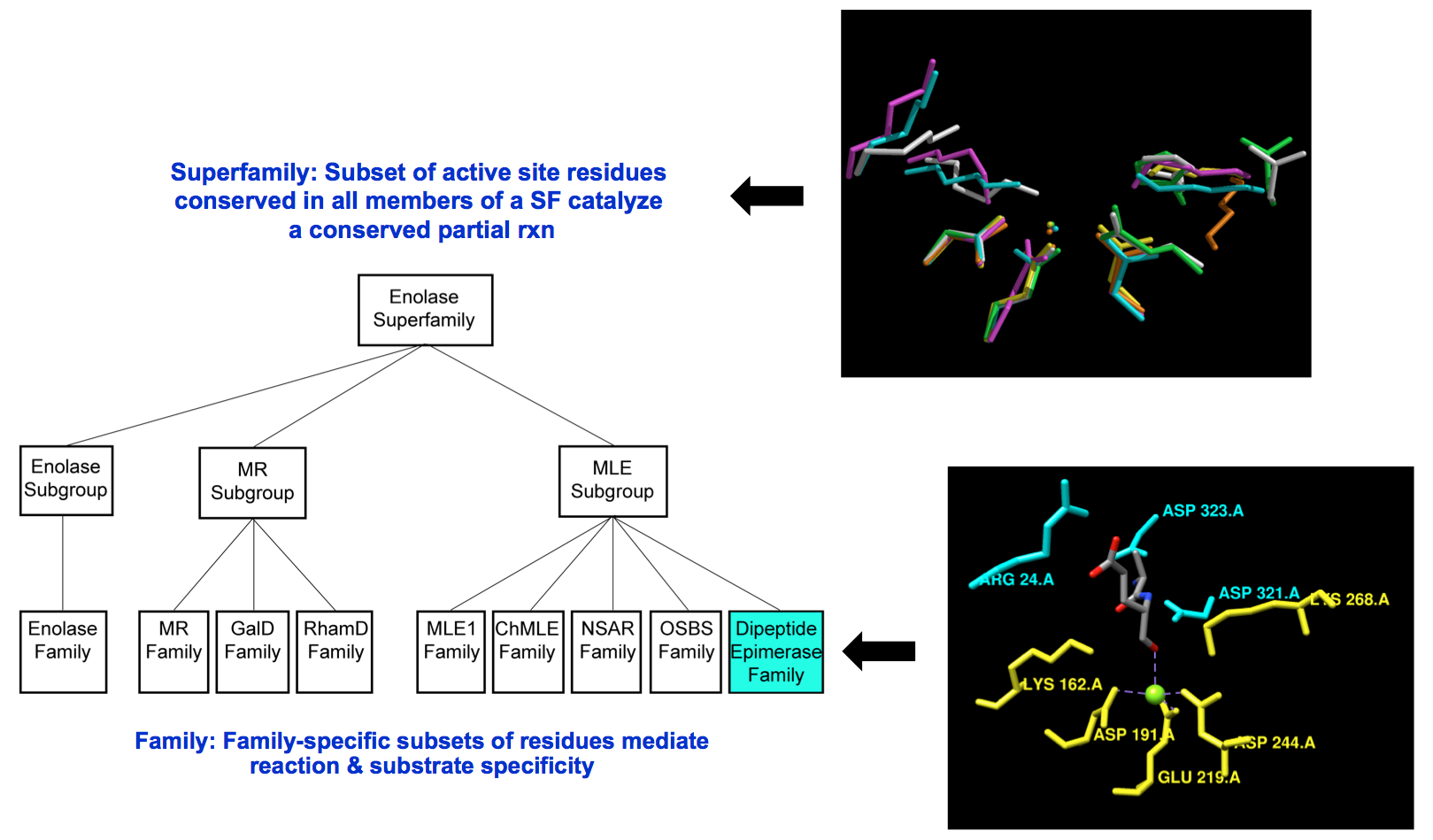

Supplement: Supplementary Data [file supp_bav063_suppl_data.zip › Fig S3 May14.png]
